# Commonalities and specialties in photosynthetic functions of PROTON GRADIENT REGULATION5 variants in Arabidopsis

**DOI:** 10.1093/plphys/kiac362

**Published:** 2022-08-10

**Authors:** Jan-Ferdinand Penzler, Giada Marino, Bennet Reiter, Tatjana Kleine, Belen Naranjo, Dario Leister

**Affiliations:** Plant Molecular Biology, Faculty of Biology, Ludwig-Maximilians-Universität München, D-82152 Planegg-Martinsried, Germany; Plant Molecular Biology, Faculty of Biology, Ludwig-Maximilians-Universität München, D-82152 Planegg-Martinsried, Germany; Plant Molecular Biology, Faculty of Biology, Ludwig-Maximilians-Universität München, D-82152 Planegg-Martinsried, Germany; Plant Molecular Biology, Faculty of Biology, Ludwig-Maximilians-Universität München, D-82152 Planegg-Martinsried, Germany; Plant Molecular Biology, Faculty of Biology, Ludwig-Maximilians-Universität München, D-82152 Planegg-Martinsried, Germany; Plant Molecular Biology, Faculty of Biology, Ludwig-Maximilians-Universität München, D-82152 Planegg-Martinsried, Germany

## Abstract

The PROTON GRADIENT REGULATION5 (PGR5) protein is required for trans-thylakoid proton gradient formation and acclimation to fluctuating light (FL). PGR5 functionally interacts with two other thylakoid proteins, PGR5-like 1 (PGRL1) and 2 (PGRL2); however, the molecular details of these interactions are largely unknown. In the Arabidopsis (*Arabidopsis thaliana*) *pgr5-1* mutant, the PGR5_G130S_ protein accumulates in only small amounts. In this work, we generated a knockout allele of *PGR5* (*pgr5-Cas*) using CRISPR-Cas9 technology. Like *pgr5-1*, *pgr5-Cas* is seedling-lethal under FL, but photosynthesis and particularly cyclic electron flow, as well as chlorophyll content, are less severely affected in both *pgr5-Cas* and *pgrl1ab* (which lacks PGRL1 and PGR5) than in *pgr5-1*. These differences are associated with changes in the levels of 260 proteins, including components of the Calvin–Benson cycle, photosystems II and I, and the NDH complex, in *pgr5-1* relative to the wild type (WT), *pgr5-Cas*, and *pgrl1ab*. Some of the differences between *pgr5-1* and the other mutant lines could be tentatively assigned to second-site mutations in the *pgr5-1* line, identified by whole-genome sequencing. However, others, particularly the more pronounced photosynthetic defects and PGRL1 depletion (compared to *pgr5-Cas*), are clearly due to specific negative effects of the amino-acid substitution in PGR5_G130S_, as demonstrated by complementation analysis. Moreover, *pgr5-1* and *pgr5-Cas* plants are less tolerant to long-term exposure to high light than *pgrl1ab* plants. These results imply that, in addition to the previously reported necessity of PGRL1 for optimal PGR5 function, PGR5 is required alongside PGRL1 to avoid harmful effects on plant performance.

## Teaser

Plants completely devoid of the PROTON GRADIENT REGULATION5 (PGR5) protein are less affected in chloroplast proteome and photosynthesis than plants with the mutated version of the protein PGR5_G130S_.

## Introduction

During photosynthesis, plants convert light energy into chemical energy (ATP and NADPH), which is subsequently used for CO_2_ fixation and other metabolic processes. The photosynthetic light reactions in plants take place in the thylakoid membranes of the chloroplast, driving linear electron flow (LEF) from water to ferredoxin (Fd) and then to NADP^+^, in a process that involves photosystems I (PSI) and II (PSII) and the cytochrome (cyt) *b*_6_*f* complex. Moreover, cyclic electron flow (CEF) around PSI also generates ATP, but does not involve PSII or produce NADPH (Alric and Johnson, 2017; Nawrocki et al., 2019). During CEF, electrons are transferred from Fd to the plastoquinone (PQ) pool, and then returned to PSI via cyt *b*_6_*f*. This latter step can occur via two different pathways. The NDH pathway is mediated by the NADH-dehydrogenase-like complex, which acts as a Fd-PQ reductase (FQR) (Yamamoto et al., 2011; Peltier et al., 2016), while the PGR5-dependent pathway (also known as the antimycin [AA]-sensitive pathway) involves the proteins PROTON GRADIENT REGULATION5 (PGR5) and PGR5-1ike 1 (PGRL1) (Munekage et al., 2002; DalCorso et al., 2008), as well as the recently discovered PGR5-1ike 2 (PGRL2) protein (Ruhle et al., 2021). However, the molecular details of this second pathway remain largely unknown.

PGR5-dependent CEF in plants not only contributes to the generation of a proton gradient (ΔpH) across the thylakoid membrane that drives ATP synthesis, but it is also essential for protection against photo-inhibition. In fact, the trans-thylakoid proton gradient both induces thermal dissipation of the excess energy in PSII through the qE component of non-photochemical quenching (NPQ) and downregulates cyt *b*_6_*f* activity (photosynthetic control), decreasing LEF and protecting PSI against excessive accumulation of electron donors (Yamori and Shikanai, 2016). Indeed, PGR5 was discovered in a screen for Arabidopsis (*Arabidopsis thaliana*) mutants that exhibited decreased quenching of chlorophyll fluorescence (Shikanai et al., 1999), and the *pgr5-1* mutant was found to be deficient in NPQ induction (Munekage et al., 2002). Moreover, *pgr5-1* also displays over-reduction of the stroma and an increase in the NADPH/ATP ratio, caused by an imbalance between LEF and CEF (Munekage et al., 2002). Consequently, *pgr5-1* plants suffer from photo-inhibition under both HL (Munekage et al., 2002; Barbato et al., 2020) and FL, which results in lethality at the seedling stage (Suorsa et al., 2012; Tikkanen et al., 2012). Strikingly, the *pgr5-1* mutation causes an amino-acid substitution (G130S) near the C-terminus, which destabilizes the protein (Munekage et al., 2002), such that only minuscule amounts of the mutant polypeptide (PGR5_G130S_) are detectable (Yamamoto and Shikanai, 2019; Barbato et al., 2020).

The PGRL1 protein interacts with PGR5, and plants that are devoid of PGRL1 (in the *pgrl1ab* mutant) fail to accumulate PGR5 and generally show a *pgr5*-like phenotype (DalCorso et al., 2008). PGRL1 contains six redox-active cysteines (Cys), and is a target for thioredoxin-mediated redox regulation of the CEF pathway (Hertle et al., 2013; Okegawa et al., 2020; Wolf et al., 2020; Naranjo et al., 2021). PGR5, however, has only one Cys residue, which is not essential for its functionality in *Chlamydomonas reinhardtii* (Buchert et al., 2020). The PGRL2 protein was recently identified as a distant homolog of PGRL1 in Arabidopsis, and it functionally interacts with both PGR5 and PGRL1 in CEF (Ruhle et al., 2021). However, in the absence of both PGRL1 and PGRL2 (in the *pgrl1ab pgrl2-1* mutant), the PGR5 protein can accumulate again, and CEF is restored (Ruhle et al., 2021). Alterations in PGR5 levels, for example, via *PGR5* overexpression, have negative effects on plant development (Okegawa et al., 2007; Long et al., 2008). Therefore, in the current model of PGR5 function, PGRL1 and PGRL2 act as antagonistic regulators of PGR5 accumulation, with PGRL1 displaying a PGR5-supporting role (Ruhle et al., 2021).

To further study the functions of and interplay between PGR5 and PGRL1, we have used CRISPR-Cas9 technology to generate an Arabidopsis *pgr5* mutant allele that is completely devoid of the PGR5 protein. Comparative analysis of this line (*pgr5-Cas*), *pgr5-1*, and plants lacking both PGR5 and PGRL1 (*pgrl1ab*) revealed that under certain conditions the PGR5_G130S_ variant has detrimental effects on photosynthesis and plant development, such that plants without PGR5 perform better than *pgr5-1* plants. Likewise, plants with PGRL1 but without PGR5 also display impairments under certain circumstances, which are not seen in plants lacking both proteins. This implies that PGR5 without PGRL1 (Ruhle et al., 2021) and PGRL1 without PGR5 can each trigger harmful effects.

## Results

### A CRISPR-Cas9-induced knockout allele of PGR5 reproduces the lethal phenotype of the original *pgr5-1* mutant under FL

**Figure 1:**
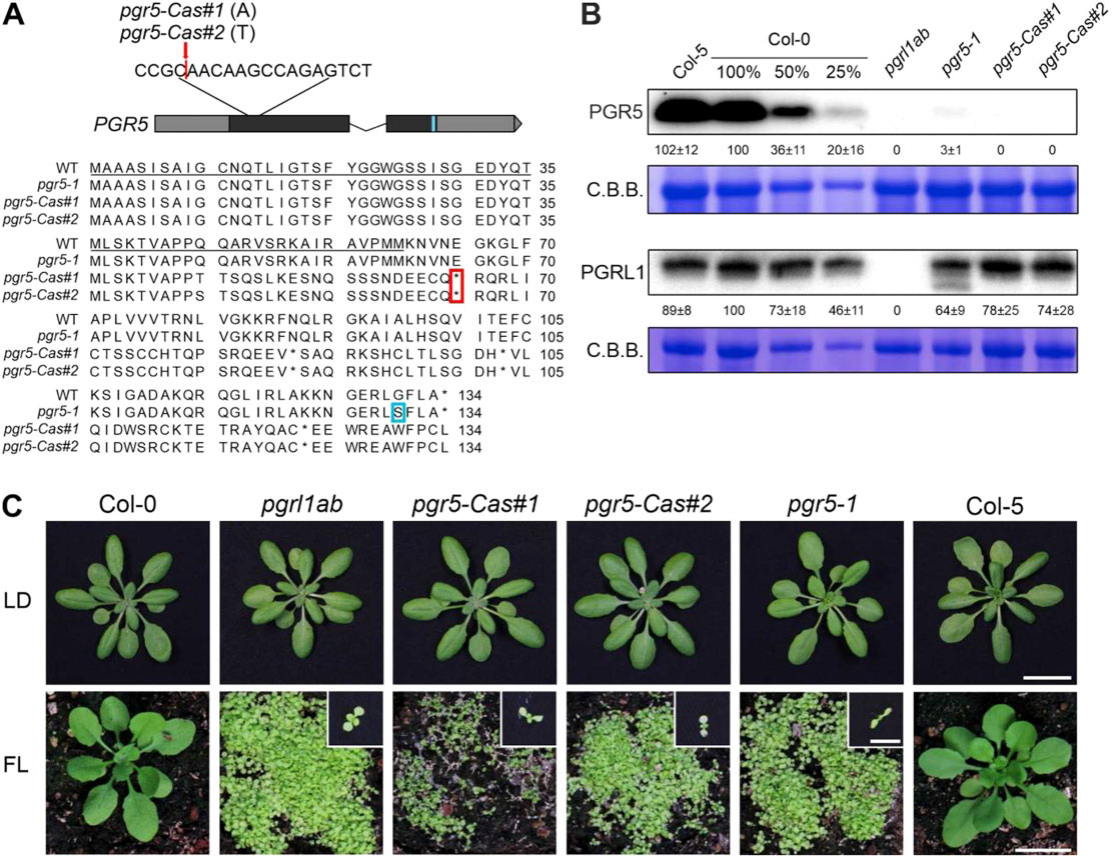
pgr5-Cas is a knock-out allele of *PGR5*. A, At the top, the Arabidopsis (*A. thaliana*) *PGR5* coding sequence is shown schematically, and the position of the nucleotide insertions in the *pgr5-Cas#1* and *#2* mutants (red) are shown, as well as the nucleotide substitution T/C in *pgr5-1* (blue). The 5′- and 3′-UTR regions are shown as gray boxes, the exons in black, and the intron as a line. At the bottom, the alignments of the encoded PGR5 protein variants in Col-0 (WT), *pgr5-1*, and *pgr5-Cas* backgrounds are shown. The red rectangle indicates the stop codon at position 65 in both *pgr5-Cas#1* and *#2* lines. The blue rectangle indicates the G130S mutation in the *pgr5-1* line. Underlined amino acids correspond to the chloroplast transit peptide sequence. B, Aliquots of total leaf proteins were isolated from Col-5, Col-0, *pgrl1ab*, *pgr5-1*, *pgr5-Cas#1*, and *pgr5-Cas#2* plants after 3 weeks of growth under LD conditions, fractionated by SDS-PAGE under reducing conditions, and subjected to immunoblotting using PGR5- or PGRL1-specific antibodies. Decreasing amounts of Col-0 were loaded as a control. Protein samples were adjusted according to fresh weight. PVDF membranes were stained with Coomassie brilliant blue (CBB) to show protein loading. Representative blots from three experiments are presented, as well as the average of the three replicates of the values corresponding to the quantification of the intensity of each band relative to Col-0 (100%) ± sd. Intensities of bands were quantified using ImageJ software (National Institutes of Health). C, Growth phenotypes of WT (Col-0 and Col-5) and mutant (*pgrl1ab*, *pgr5-Cas#1*, *pgr5-Cas#2*, and *pgr5-1*) plants propagated for 3 weeks under LD (16-h light/8-h dark, 100 µmol photons m^−2^ s^−1^) or 4 weeks under FL (12-h light/12-h dark, with cycles of 50 µmol photons m^−2^ s^−1^ for 5 min and 500 µmol photons m^−2^ s^−1^ for 1 min during the light period) conditions. In both LD and FL conditions, plants were sown at high density and then separated. The scale bars at the bottom of LD and FL images correspond to 2 and 0.5 cm for the inset images. The scale bars refer to all images of the same condition.

To create a knock-out allele of *PGR5*, we employed the CRISPR-Cas9 technology and generated the lines *pgr5-Cas#1* and *#2* by inserting an extra nucleotide (“A” for *pgr5-Cas#1* and “T” for *pgr5-Cas#2*) between positions 132 and 133 of *PGR5* (Figure 1A). Each of these insertions produces a premature stop codon just five amino acids downstream of the transit peptide sequence (Figure 1A), which completely suppresses expression of the PGR5 protein in the *pgr5-Cas* mutants, as confirmed by Western blot analysis (Figure 1B and Supplemental Figure S1A). In the original *pgr5* mutant, *pgr5-1* (Munekage et al., 2002), which carries an amino-acid substitution (G130S) at the C-terminus of the protein (Figure 1A), accumulation of PGR5_G130S_ is strongly impaired, but small amounts of the mutant protein are still detectable (Yamamoto and Shikanai, 2019; Barbato et al., 2020); in our hands, they correspond to about 3% of the WT level (Figure 1B and Supplemental Figure S1A). Furthermore, the PGRL1 content appeared to be more decreased in the *pgr5-1* mutant compared with the *pgr5-Cas* lines. In immunoblots probed with a PGRL1-specific antibody, a band of lower molecular weight corresponding to 13% of the WT content of PGRL1 was detectable exclusively in the *pgr5-1* background, possibly indicating the accumulation of degradation products of PGRL1 in this genotype (Figure 1B).

Under long-day (LD) growth conditions, *pgr5-Cas* plants displayed *pgr5-1*-like growth (Figure 1C), but under FL, they died at the seedling stage, like *pgr5-1* (Suorsa et al., 2012; Tikkanen et al., 2012) and *pgrl1ab* (Ruhle et al., 2021; Figure 1C).

### The two *pgr5* mutants and *pgrl1ab* differ subtly in their photosynthetic performance

We then asked whether the total lack of PGR5 in *pgr5-Cas* has a stronger effect on CEF than that seen in *pgr5-1*. To this end, we monitored the photosynthetic performance of these plants and determined several parameters which can be affected by CEF, such as maximum transient NPQ (tNPQ), P700 oxidation, and electrochromic shifts (ECS).

**Figure 2:**
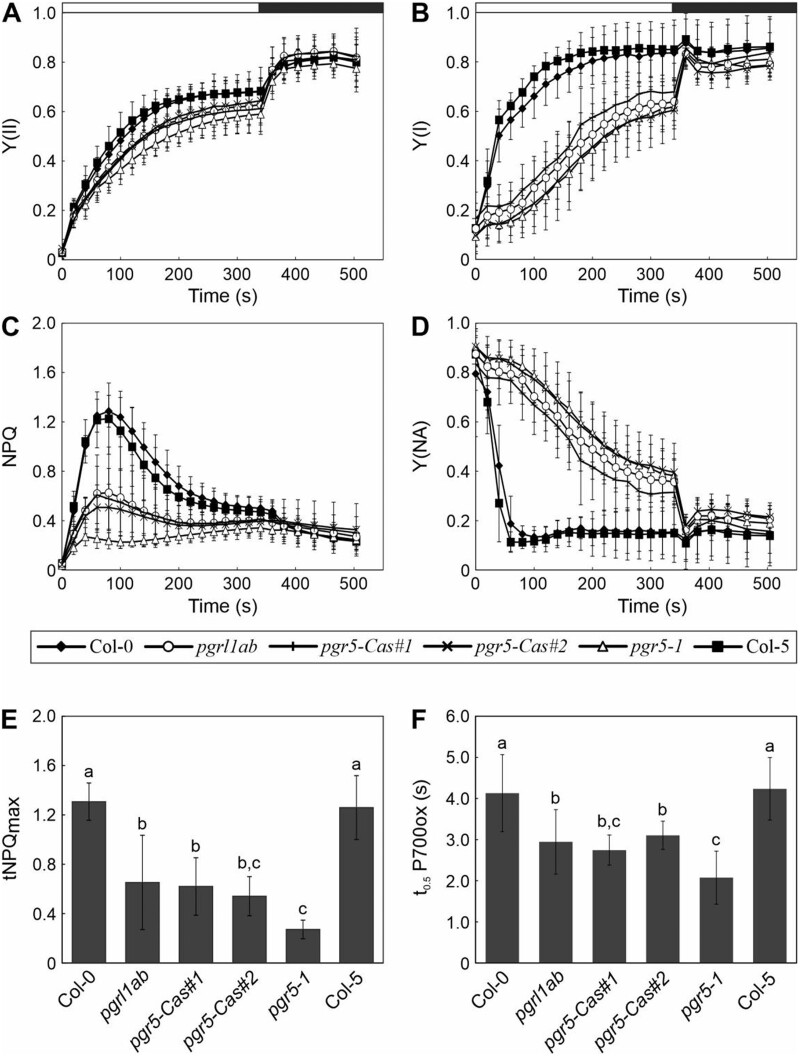
Photosynthetic performance and assessment of CEF rates in the different genotypes. A, PSII quantum yield (Y(II)) was determined based on chlorophyll fluorescence monitored in dark-adapted WT (Col-0 and Col-5) and mutant (*pgrl1ab*, *pgr5-Cas#1*, *pgr5-Cas#2*, and *pgr5-1*) plants grown under LD conditions for 3 weeks. Plants were illuminated for 6 min with actinic light (100 μmol photons m^−2^ s^−1^, white bar), followed by a dark period of 3 min (black bar). Saturating pulses were applied every 20 s. B, PSI quantum yield (Y(I)) was determined based on absorbance measurements at 830 versus 875 nm in the same plants as in (A) and following the same induction-recovery and saturation-pulse analysis. C, NPQ was determined during the measurements shown in (A). D, PSI acceptor-side (Y(NA)) imitation was determined during the measurements shown in (B). E, Average-tNPQ_max_ values obtained in (B) in the first seconds after illumination. F, Same plants as in (A) were subjected to 5 s of actinic light (660 μmol photons m^−2^ s^−1^), followed by 2 s of dark, and then 23 s of FR light. P700 oxidation was monitored during the last FR period as the difference between the transmittance signals at 830 and 875 nm, respectively. The time taken to reach half oxidation of P700 (t_0.5_ P700ox) is shown for each genotype. Averages of at least eight plants from two independent experiments are shown. Error bars represent standard deviations. Different letters above error bars represent statistical difference (*P* < 0.05) as determined by Tukey’s test.

When photosynthesis was analyzed following exposure of dark-adapted plants to 100 µmol photons m^−2^ s^−1^ of actinic light (similar to the flux provided during their growth) and recovery in the dark, we found that in general all mutant genotypes behaved similarly with respect to the quantum yields of PSII (Y(II)) and PSI (Y(I)), NPQ, and PSI acceptor-side limitation (Y(NA)) (Figure 2), as well as non-regulated energy dissipation quantum yield (Y(NO)) and PSI donor-side limitation (Y(ND)) (Supplemental Figure S2). Nevertheless, *pgr5-1* plants were always slightly more impaired with respect to the measured photosynthetic parameters than *pgr5-Cas* and *pgrl1ab* plants (Figure 2; Supplemental Figure S2 and Supplemental Table S1). Interestingly, we observed that the photosynthetic phenotype in the *pgrl1ab* mutant, which also lacks PGR5 (DalCorso et al., 2008), was more similar to that of the *pgr5-Cas* lines than that of the *pgr5-1* mutant (Figure 2 and Supplemental Figure S2). Thus, analyzing in more detail parameters that can be affected by CEF, we observed that in the two *pgr5-Cas* lines, as well as in *pgrl1ab*, they were less severely affected than in *pgr5-1* (Figure 2, E and F and Supplemental Figure S2C). In the case of the tNPQ_max_ and the time needed to oxidize 50% of P700 (t_0.5_P700ox), the differences between *pgr5-1* and *pgrl1ab* plants were statistically significant, while the *pgr5-Cas* lines were similar to *pgrl1ab*, although not always statistically different to *pgr5-1* (Figure 2, E and F and Supplemental Tables S1 and S2). In addition, the differences in the tNPQ_max_ values between *pgr5-1* and *pgrl1ab*, as well as the two *pgr5-Cas* lines, were also observed at the seedling stage of development (Supplemental Figure S3) and when plants were grown under low-light conditions (50 µmol photons m^−2^ s^−1^) (Supplemental Figure S4C). A similar trend with respect to the differences between genotypes was observed for the electron transport rate of PSII (ETR (II)) and NPQ under stepwise increase of light intensity (Supplemental Figure S5), as well as for NPQ after a transition from dark to different light intensities (110, 280, or 530 µmol photons m^−2^ s^−1^ of actinic light), with *pgr5-1* always displaying the lowest values (Supplemental Figure S6).

Together, these data suggest that knock-out of *PGR5* (in *pgr5-Cas*) results in effects that are generally similar to those seen in *pgr5-1* and *pgrl1ab*, while the *pgr5-1* mutant, which expresses very low levels of the PGR5_G130S_ variant, displays more severe impairments of photosynthesis—especially in PSII parameters—than plants that lack PGR5 (*pgr5-Cas*) or both PGR5 and PGRL1 (*pgrl1ab*).

### Chloroplast protein composition is markedly altered in the *pgr5-1* mutant, but not in *pgrl1ab* and *pgr5-Cas* plants

**Figure 3:**
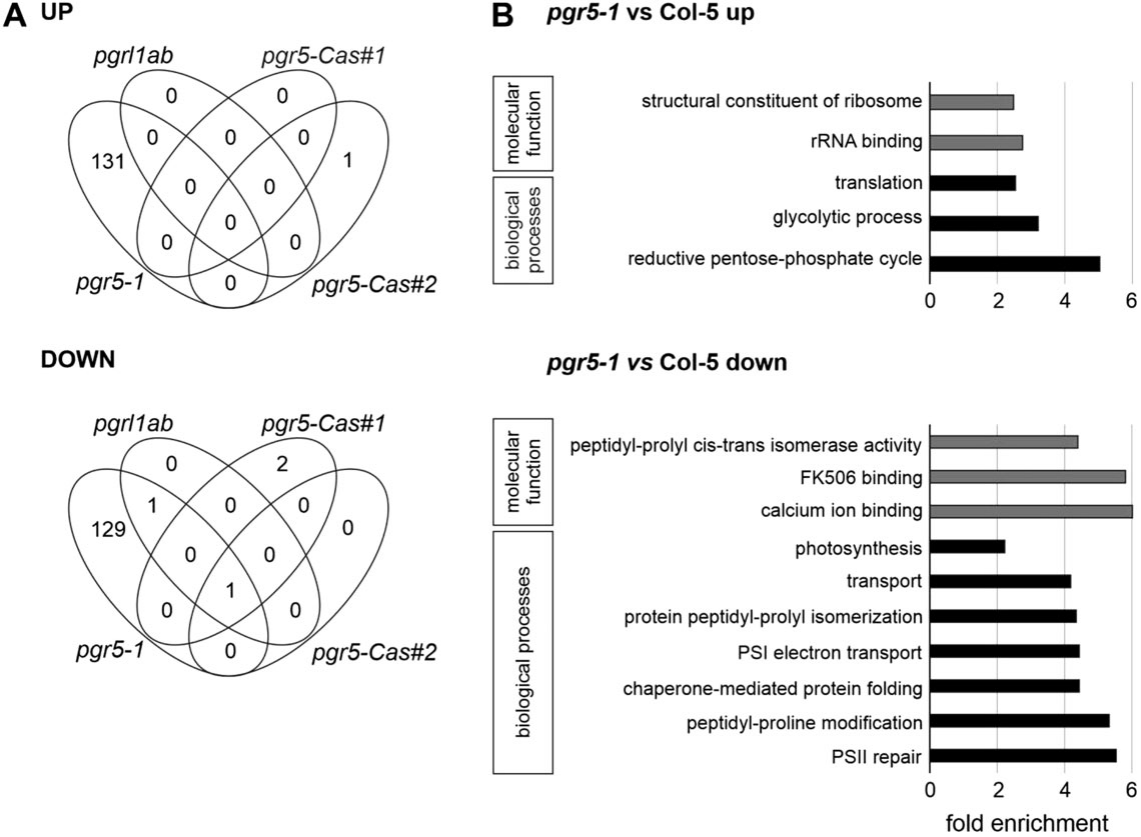
Comparative proteomic analysis of *pgr5* (*pgr5-1*, *pgr5-Cas#1*, and *pgr5-Cas#2*) and *pgrl1ab* mutants. A, Venn diagrams depict the numbers of statistically significant up- and down-regulated (1.5-fold up or down in comparison to the WT and with FDR < 0.05, *n* = 3) proteins in comparison to Col-5 (*pgr5-1*) or Col-0 (*pgr5-Cas#1*, *pgr5-Cas#2*, and *pgrl1ab*). B, Gene ontology (GO) analysis of chloroplast proteins whose expression was up- or down-regulated in *pgr5-1* compared with WT (Col-5). GO annotations for the biological process (gray bars) and molecular function (black bars) categories were extracted from DAVID (Huang da *et al.*, 2009). GO terms with a Benjamini-corrected value of *P *<* *0.05 are plotted together with the fold enrichment for each term.

To study the molecular basis for the alterations in photosynthesis observed in *pgr5-1* relative to *pgr5-Cas* and *pgrl1ab*, we analyzed the chloroplast proteomes of these lines, employing two different WT controls (Col-0 or Col-5, depending on the mutant background concerned). This analysis showed that the *pgrl1ab* and *pgr5-Cas* mutations had almost no effect on the protein content compared with WT (Col-0) plants. In *pgrl1ab*, only two proteins were down-regulated relative to Col-0, namely PGRL1 itself (as in *pgr5-1*, but not in *pgr5-Cas*) and PGR5 (as in all of the mutants analyzed) (Figure 3A and Supplemental Table S3). In the two *pgr5-Cas* mutants, only a few chloroplast proteins were differentially expressed to a significant degree (DEPs), and only PGR5 itself was missing in both *pgr5-Cas*#1 and #2. In addition, only the Calvin–Benson cycle protein CP12-1 (At2g47400) and a tRNA dimethylallyltransferase (At5g52960) were downregulated in *pgr5-Cas#1*, and only the Initiation Factor 4F subunit (At3g43540) was upregulated in *pgr5-Cas#2* (Figure 3A and Supplemental Table S3).

By striking contrast, in the *pgr5-1* line a total of 262 significant DEPs compared with Col-5 were identified, 131 of which were up- (fold ratio > 1.5) and 131 down-regulated (fold ratio < 0.66; Figure 3A and Supplemental Table S3). As mentioned above, among the latter were PGR5 (which is also down-regulated in the other genotypes) and PGRL1 (which is down-regulated in *pgrl1ab* as well), such that no <260 DEPs are specific for the *pgr5-1* line. The DEPs found in *pgr5-1* were assigned to different categories based on their molecular functions and the biological processes in which they are involved (Figure 3B). This classification reveals that the reductive pentose-phosphate cycle/Calvin cycle is up-regulated, whereas photosynthesis/light reactions are down-regulated—in particular, PSI electron transport and PSII repair (Figure 3B). Indeed, a closer look at the individual DEPs shows that various thylakoid proteins are down-regulated in *pgr5-1*, including components of the ATP synthase, cyt *b*_6_*f*, the NDH complex, PSI, and PSII, while proteins that participate in the Calvin–Benson cycle are up-regulated (Table 1). Some proteins involved in chlorophyll biosynthesis were also more abundant in *pgr5-1* than in WT (Table 1). However, the total amount of chlorophyll in *pgr5-1* was lower than in the WT and the other mutants analyzed (Supplemental Table S4).

**Table 1:** List of selected statistically significant changing proteins related to photosynthesis identified in the *pgr5-1* versus Col-5 proteomic experiment

Protein IDs	Gene name	Description	Ratio *pgr5-1*/Col-5	Adjusted *P*-value
Calvin–Benson cycle				
P25851	*CFBP1*	Fructose-1,6-bisphosphatase 1 (FBPase)	2.731	3.99*E*−03
P25856	*GAPA1*	Glyceraldehyde 3-P dehydrogenase A sub1	2.382	3.53*E*−03
Q9LPW0; F4HNZ6	*GAPA2*	Glyceraldehyde 3-P dehydrogenase A sub2	2.402	3.53*E*−03
P25857	*GAPB*	Glyceraldehyde-3-P dehydrogenase B	2.612	3.53*E*−03
Q9LD57	*PGK1*	Phosphoglycerate kinase 1	1.905	3.53*E*−03
P50318; Q9SAJ4	*PGK2*	Phosphoglycerate kinase 2; 3	1.504	2.17*E*−02
P10795; F4HRR5	*RBCS-1A*	Rubisco small chain 1A	3.672	3.53*E*−03
P10796	*RBCS-1B*	Rubisco small chain 1B	2.841	3.53*E*−03
P10798; P10797	*RBCS-2B*	Rubisco small chain 2B	4.211	3.53*E*−03
P10896	*RCA*	Rubisco activase	2.226	3.53*E*−03
P46283	*SBPASE*	Sedoheptulose-bisphosphatase	2.156	3.53*E*−03
Q9SKP6	*TIM*	Triosephosphate isomerase	2.251	4.24*E*−03
Q8RWV0; F4IW47	*TKL-1; TKL-2*	Transketolase1; 2	2.280	3.53*E*−03
PSI				
P56766	*psaA*	PSI chlorophyll a apoprotein A1	0.116	1.56*E*−02
P56767	*psaB*	PSI chlorophyll a apoprotein A2	0.374	3.34*E*−02
Q9S831	*PsaE1*	PSI reaction center subunit IV A	0.602	2.84*E*−02
Q9S714	*PsaE2*	PSI reaction center subunit IV B	0.595	2.36*E*−02
Q9S7N7	*PsaG*	PSI reaction center subunit V	0.625	4.57*E*−02
Q949Q5	*PsaO*	PSI subunit O	0.628	7.40*E*−03
Q8LCA1	*PsaP/CURT1B*	Curvature thylakoid 1B	0.498	3.53*E*−03
PSII				
P56777	*psbB*	PSII CP47 reaction center protein	0.676	5.89*E*−03
P56779	*psbE*	Cyt b559 subunit alpha	0.659	7.42*E*−03
P62095	*psbF*	Cyt b559 subunit beta	0.513	3.62*E*−03
P56780	*psbH*	PSII reaction center protein H	0.343	4.72*E*−02
Q9S841	*PSBO2*	Oxygen-evolving enhancer protein 1-2	0.544	4.66*E*−03
Q9XFT3	*PSBQ1*	Oxygen-evolving enhancer protein 3-1	0.658	1.52*E*−02
P27202	*PsbR*	PSII 10 kDa polypeptide	0.611	4.64*E*−03
Q39195	*PsbTn*	PSII 5 kDa protein	0.537	1.90*E*−02
Antenna PSI and PSII				
Q9C639	*LHCA5/CURT1C*	Chlorophyll a/b-binding protein 5	0.341	3.53*E*−03
Q8VZ87; P0CJ48	*LHCB1.1; 1.2*	Chlorophyll a-b binding protein 2; 3	2.639	2.81*E*−02
Q07473	*LHCB4.1*	Chlorophyll a-b binding protein CP29.1	0.656	1.07*E*−02
Q9XF88	*LHCB4.2*	Chlorophyll a-b binding protein CP29.2	0.608	1.03*E*−02
PGRL1/PGR5—CEF component				
Q9SL05	*PGR5*	Protein PGR5	0.015	3.77*E*−05
Q8H112	*PGRL1A*	PGR5-like protein 1A	0.532	4.60*E*−03
NDH complex				
P56755	*ndhI*	NAD(P)H-quinone oxidoreductase sub I	0.660	1.03*E*−02
Q2V2S7	*NDHM*	NAD(P)H-quinone oxidoreductase sub M	0.449	3.53*E*−03
Q9LVM2	*NDHN*	NAD(P)H-quinone oxidoreductase sub N	0.613	1.40*E*−02
Q84VQ4	*NDHU/CRRL*	NAD(P)H-quinone oxidoreductase sub S	0.479	3.53*E*−03
Q9T0A4	*NDHS/CRR31*	NAD(P)H-quinone oxidoreductase sub U	0.593	3.53*E*−03
Q94AQ8	*PNSB2/NDH45*	NDH subunit of subcomplex B2	0.376	4.54*E*−02
Q9LU21	*PNSB3/NDF4*	NDH subunit of subcomplex B3	0.608	1.02*E*−02
Q8RXS1	*PNSB4/NDF6*	NDH subunit of subcomplex B4	0.494	2.82*E*−02
O80634	*PNSL1/PPL2*	NDH subunit of lumenal location 1	0.377	3.53*E*−03
Q9XI73	*PNSL2/PSBQ-F1*	NDH subunit of lumenal location 2	0.402	3.53*E*−03
Q9SGH4	*PNSL3/PSBQ-F2*	NDH subunit of lumenal location 3	0.538	3.73*E*−03
Q9SCY3	*PNSL4/FKB16-2*	NDH subunit of lumenal location 4	0.623	1.60*E*−02
Cyt *b*_6_*f*				
P56771	*petA*	Cyt f	0.676	7.52*E*−03
P56773	*petB*	Cyt b6	0.621	2.90*E*−02
PSI biogenesis				
Q9SLI4	*RBD1*	Rubredoxin-like superfamily protein	0.523	1.05*E*−02
O23403	*PPD1*	PsbP domain-containing protein 1	0.589	5.35*E*−03
Q9LU01	*Y3IP1*	Ycf3-interacting protein 1	0.665	1.27*E*−02
Q6STH5	*HCHF101*	Fe-S cluster assembly factor HCF101	1.504	1.80*E*−02
PSII biogenesis				
Q9SRY4	*LPA1*	Protein LOW PSII ACCUMULATION 1	0.322	3.53*E*−03
Q9LR64	*PSB27*	PSII repair protein PSB27-H1	0.460	3.53*E*−03
Q9ZVL6	*TLP18.3*	Thylakoid Lumen Protein 18.3	0.493	3.53*E*−03
Q39102	*FTSH1*	Zinc metalloprotease FTSH 1	0.500	1.56*E*−02
Q8W585; Q9M895	*FTSH8/FTSHI3*	Zinc metalloprotease FTSH 8/FTSHI 3	0.530	5.15*E*−03
Q9LU10	*DEG8*	Protease Do-like 8	0.532	3.53*E*−03
Q9ZVZ9	*LPA19*	PSII D1 precursor processing protein	0.539	4.80*E*−03
O22609	*DEG1*	Protease Do-like 1	0.587	3.53*E*−03
O80860	*FTSHH2*	Zinc metalloprotease FTSH 2	0.642	1.85*E*−02
P82538	*PPL1*	PsbP-like protein 1	0.662	4.64*E*−03
P34791	*CYP20-3*	Peptidyl-prolyl cis-trans isomerase	2.586	5.15*E*−03
Cyt *b*_6_*f* biogenesis				
O23166	*HCF164*	Thioredoxin-like protein HCF164	0.514	3.53*E*−03
Q94BY7	*DAC*	Defective Aaccumulation of Cyt b6/f	0.521	3.99*E*−03
Q9FJ81	*CCB2*	Cofactor Assembly of Complex C subunit B	0.668	6.62*E*−03
Chlorophyll biosynthesis				
Q9LR75; Q93Z96	*HEMF1; 2*	Coproporphyrinogen-III oxidase 1; 2	1.504	2.04*E*−02
Q9FNB0	*CHLH*	Magnesium-chelatase subunit ChlH	1.600	1.85*E*−02
P16127	*CHLI*	Magnesium-chelatase subunit ChlI-1	1.885	7.40*E*−03
Q43316	*HEMC*	Porphobilinogen deaminase	1.907	4.22*E*−03
Others				
Q42139	*ATPG*	ATPase, F0 complex, subunit B/B'	0.665	3.13*E*−02
O04616; B3H429	*CURT1A*	Curvature Thylakoid 1A	0.570	5.86*E*−03
Q8LDD3	*CURT1D*	Curvature Thylakoid 1D-like protein	0.626	1.45*E*−02
O64730	*PBCP*	Protein phosphatase 2C 26	0.322	2.00*E*−02

Taken together, the marked differences in chloroplast protein content between *pgr5-1* and *pgrl1ab* or *pgr5-Cas* could account for the pronounced defects in photosynthesis and particularly in CEF seen in *pgr5-1*.

### The *pgr5-1* mutant harbors a set of second-site mutations affecting PSII

In principle, the changes in chloroplast protein content in the *pgr5-1* mutant described above could be caused either by additional negative effects of the residual amount of PGR5_G130S_ it expresses, or by additional second-site mutations in the *pgr5-1* line. Indeed, the fact that the *pgr5-1* mutation was induced with EMS (Shikanai et al., 1999) argues in favor of the latter explanation. We therefore sequenced the whole genome of *pgr5-1* and compared it with that of the parental background Col-5, and to Col-0. In total, we found one alteration in a splicing site and 48 non-synonymous mutations in exon regions of different genes (Supplemental Table S5), of which 5 were in genes coding for chloroplast proteins (Table 2). These five genes comprised, as expected, *PGR5* and, in addition, *PSBO2* and *HCF136*, which code for a subunit of the oxygen-evolving complex (Yi et al., 2005; Lundin et al., 2007) and a PSII assembly factor, respectively (Meurer et al., 1998). Both PsbO_2_ and HCF136 were also found to be down-regulated in our proteomic analysis (PsbO_2_ by about 50% [see also Table 1] and HCF136 by about 30%). The other two mutations were in *CGL20A*, which codes for a splicing factor (Reiter et al., 2020), and *ABCI11*, coding for an ATPase-coupled transmembrane transporter (Voith von Voithenberg et al., 2019), but neither CGL20A nor ABCI11 was detected in our proteomic analysis (Table 2 and Supplemental Table S3). Therefore, some *pgr5-1*-specific changes in the chloroplast proteome (at least the one found for PsbO_2_, Tables 1 and 2) may be attributable to the single-nucleotide polymorphisms (SNPs) identified and it is conceivable that these second-site mutations contribute to the observed *pgr5-1*-specific photosynthetic defects. However, owing to the large number of additional mutations, it is impossible to unambiguously assign changes in the abundance of individual proteins to individual SNPs. To clarify this point, we chose instead to complement the *pgr5-1* mutation itself.

**Table 2:** Chloroplast proteins associated with non-synonymous coding SNPs in the *pgr5-1* background and the associated proteomic result

			SNPs analysis		Proteomics	
Gene	Symbol	Name	nt	AA (pos)	Ratio *pgr5*-*1*/Col-5	Adjusted *P*-value
At2g05620	*PGR5*	PGR5	T/C	G/S (130)	0.015	3.77*E*−05
At2g17240	*CGL20A*	Arginine/serine-rich-like splicing factor	T/C	P/L (93)	ND	ND
At3g50820	*PSBO2*	PSII subunit O-2	T/G	P/Q (203)	0.544	4.66*E*−03
At5g14100	*ABCI11*	ABC transporter I family member 11	A/G	A/V (20)	ND	ND
At5g23120	*HCF136*	PSII stability/assembly factor	A/G	R/K (357)	0.696	1.27*E*−02

nt, nucleotide; AA, amino acid; pos, position; ND, not detected.

### Complementation analysis implies that the residual PGR5_G130S_ present in *pgr5-1* has specific effects on photosynthesis

**Figure 4:**
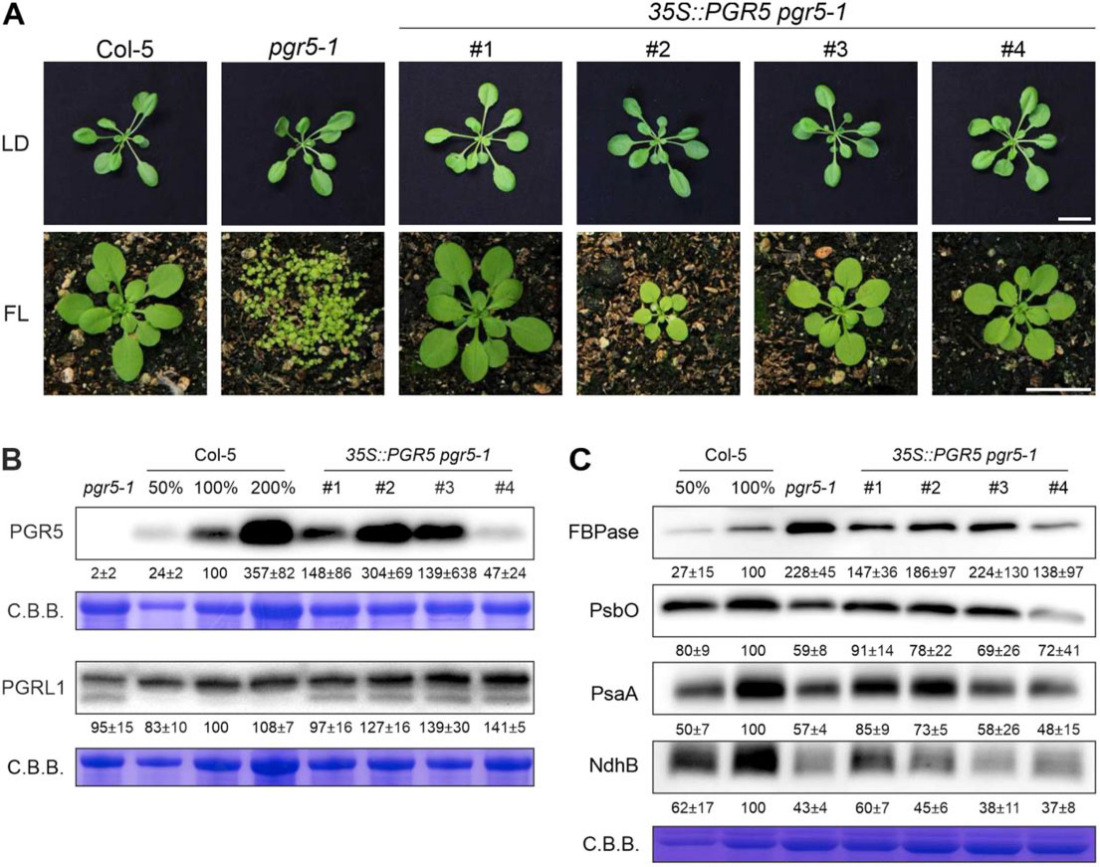
Complementation of the *pgr5-1* mutant. A, Growth phenotypes of plants (WT [Col-5], *pgr5-1* and four different *pgr5-1* lines overexpressing PGR5 [*35S*::*PGR5 pgr5-1*, #1-4]) grown under LD or FL conditions for 3 or 4 weeks, respectively. In both LD and FL conditions, plants were sown at high density and then separated. The scale bars at the bottom of LD and FL images indicate 2 cm and refer to all images of the same condition. B, Aliquots of total leaf proteins from Col-5, *pgr5-1*, and *35S::PGR5 pgr5-1* (#1-4) plants grown for 3 weeks under LD conditions were fractionated by SDS-PAGE under reducing conditions, and subjected to immunoblotting using PGR5- or PGRL1-specific antibodies. Varying amounts of Col-5 were loaded as indicated. Protein samples were adjusted according to fresh weight. PVDF membranes were stained with CBB to show protein loading. C, Immunoblot analysis of representative chloroplast proteins. Chloroplasts were isolated from Col-5, *pgr5-1*, and *35S::PGR5 pgr5-1* (#1-4) plants after 7 weeks of growth under SD conditions. Protein samples were fractionated by SDS-PAGE and subjected to immunoblotting using the indicated antibodies specific for proteins of the Calvin–Benson cycle: (FBPase), PSI (PsaA), PSII (PsbO), and the NDH complex (NdhB). Decreasing amounts of Col-5 were loaded. PVDF membranes were stained with CBB as loading control. Representative blots from three experiments are presented, as well as the average of the three replicates of the values corresponding to the quantification of the intensity of each band relative to Col-5 (100%) ± sd.

**Figure 5:**
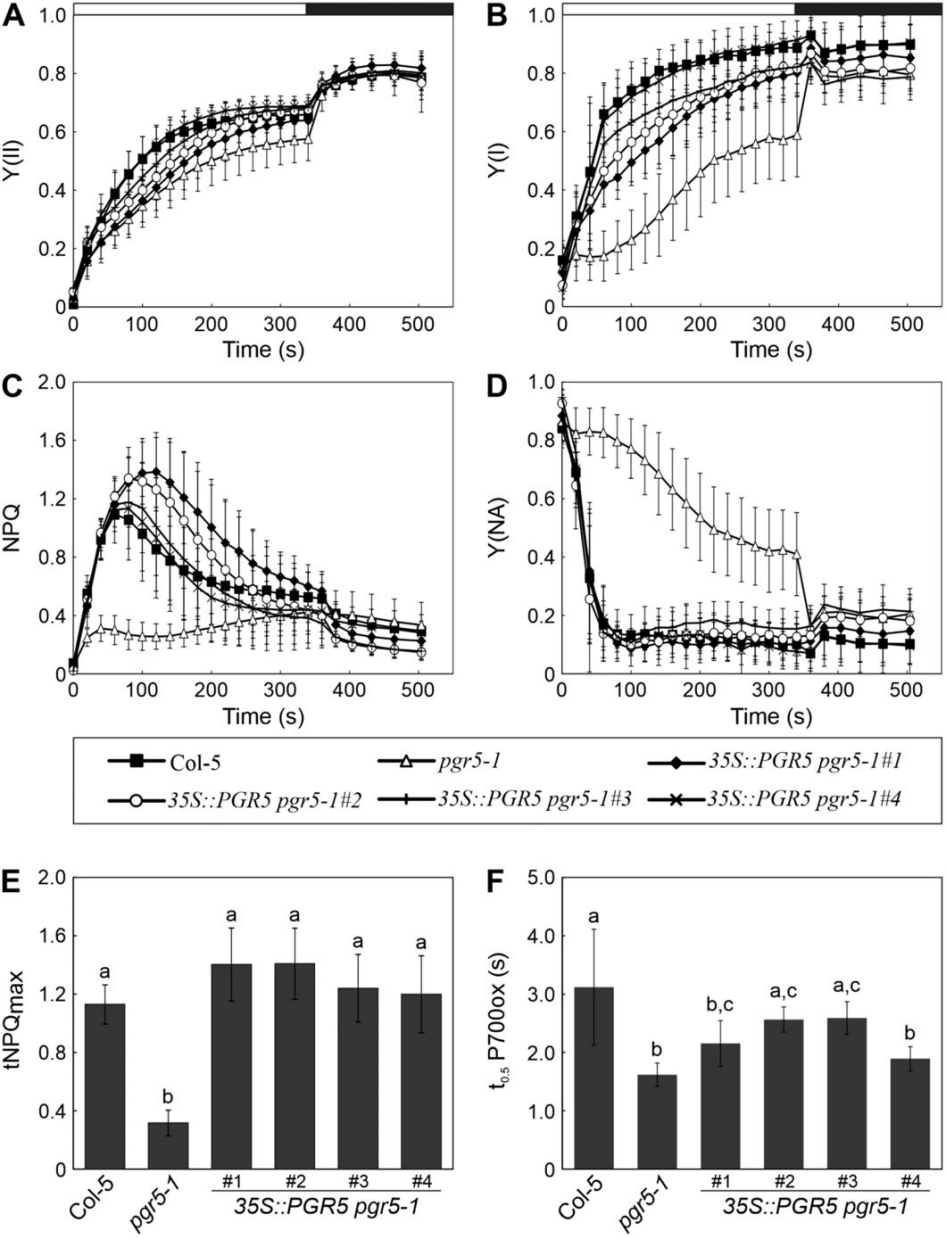
Photosynthetic performance and assessment of CEF rates in different *pgr5-1* lines complemented with WT *PGR5*. A, PSII quantum yield (Y(II)) was determined based on chlorophyll fluorescence monitored in Col-5, *pgr5-1*, and *35S::PGR5 pgr5-1* (#1-4) dark-adapted plants grown under LD conditions for 3 weeks. Plants were illuminated for 6 min with actinic light (100 μmol photons m^−2^ s^−1^, white bar), followed by a dark period of 3 min (black bar). Saturating pulses were applied every 20 s. B, PSI quantum yield (Y(I)) was determined based on absorbance measurements at 830 versus 875 nm in the same plants as in (A) and following the same induction-recovery and saturation-pulse analysis. C, NPQ was determined during the measurements shown in (A). D, PSI acceptor-side (Y(NA)) imitation was determined during the measurements shown in (B). E, Average-tNPQ_max_ values obtained in (B) in the first seconds after illumination. F, Same plants as in (A) were subjected to 5 s of actinic light (660 μmol photons m^−2^ s^−1^), followed by 2 s of dark, and then 23 s of FR light. P700 oxidation was monitored during the last FR period as the difference between the transmittance signals at 830 and 875 nm, respectively. The time taken to reach half oxidation of P700 (t_0.5_ P700ox) is shown for each genotype. Averages of at least seven plants are shown from two independent experiments. Error bars represent standard deviations. Different letters above error bars represent statistical difference (*P* < 0.05) as determined by Tukey’s test.

To directly test the impact of the additional mutations in the *pgr5-1* mutant on the overall phenotype of this line, we studied four independent lines in which PGR5 was overexpressed in the *pgr5-1* background (*35S::PGR5 pgr5-1* #1-4). All of these lines grew like WT plants under LD conditions and managed to complement the lethal phenotype of *pgr5-1* under FL to some extent (Figure 4A). Interestingly, the smallest plant under FL was the one with the highest amount of PGR5, although, in general, the growth behavior did not entirely correspond to the amount of PGR5 (Figure 4, A and B and Supplemental Figure S1B). Moreover, overexpression of PGR5 in *pgr5-1* did not reconstitute the WT chloroplast proteome in any of the four independent complemented lines (Figure 4C and Supplemental Figure S1C), most notably in the case of PsbO, PsaA, and NdhB—which implies that these effects could be attributable to second-site mutations. By contrast, in the four complementation lines PGRL1 levels were restored to WT levels, indicating that the decrease in PGRL1 abundance observed in *pgr5-1* (but not in *pgr5-Cas*) is due to the specific nature of the *pgr5-1* mutation (Figure 4B and Supplemental Figure S1B). However, the band of lower molecular weight, which may derive from the accumulation of degradation products of PGRL1 in the *pgr5-1* background (see Figure 1B), persisted in the complementation lines. This effect may therefore result either from the continuing production of the PGR5_G130S_ protein which cannot be counteracted by high levels of WT PGR5, or from a second-site mutation. Moreover, the photosynthetic parameters, measured as in Figure 2, were also analyzed for the overexpressor lines. The efficiencies of both photosystems (Y(II) and Y(I)) were partially recovered in the lines *35S::PGR5 pgr5-1* #1-3 and reached WT levels in the line #4, which had the lowest PGR5 amount (Figure 5, A and B and Supplemental Table S6). Additionally, the level of the tNPQ_max_, as well as the PSI acceptor-side limitation (Y(NA)), were completely restored to WT levels in all the lines (Figure 5, C–E and Supplemental Table S6). Moreover, the impaired tNPQ_max_ in *pgr5-1* was also partially or fully complemented in the lines *35S::PGR5 pgr5-1* #1 and #2 growing at different light intensities (Supplemental Figure S4C). Finally, the time needed to oxidize 50% of P700 (t_0.5_P700ox) was also almost restored to WT levels in two lines (#2 and #3) (Figure 5F and Supplemental Table S7). The differences in the recovery of the photosynthetic parameters between the four overexpression lines could be due to a dosage effect, as previously suggested when PGR5 was overexpressed in a WT background, which has negative effects on plant development (Okegawa et al., 2007). However, in our case, the observed effects did not entirely correspond with the PGR5 accumulation detected in Figure 4B.

In conclusion, since expression of WT-like PGR5 levels in the *pgr5-1* background cures only the effects of the *pgr5-1* mutation but not of the second-site mutations, the full restoration of NPQ (see Figure 5, C and E) indicates that the most severe photosynthetic effect observed in *pgr5-1* relative to *pgr5-Cas* or *pgrl1ab* is indeed associated with the specific nature of this mutation. The effects of the *pgr5-1* mutation should in principle be abrogated in the four *35S::PGR5 pgr5-1* lines, since the WT form of the PGR5 protein massively prevails over the mutated one, PGR5_G130S_. Thus, all remaining phenotypic deviations should be attributable to the second-site mutations.

### In the absence of PGR5, PGRL1 becomes harmful under HL conditions

**Figure 6:**
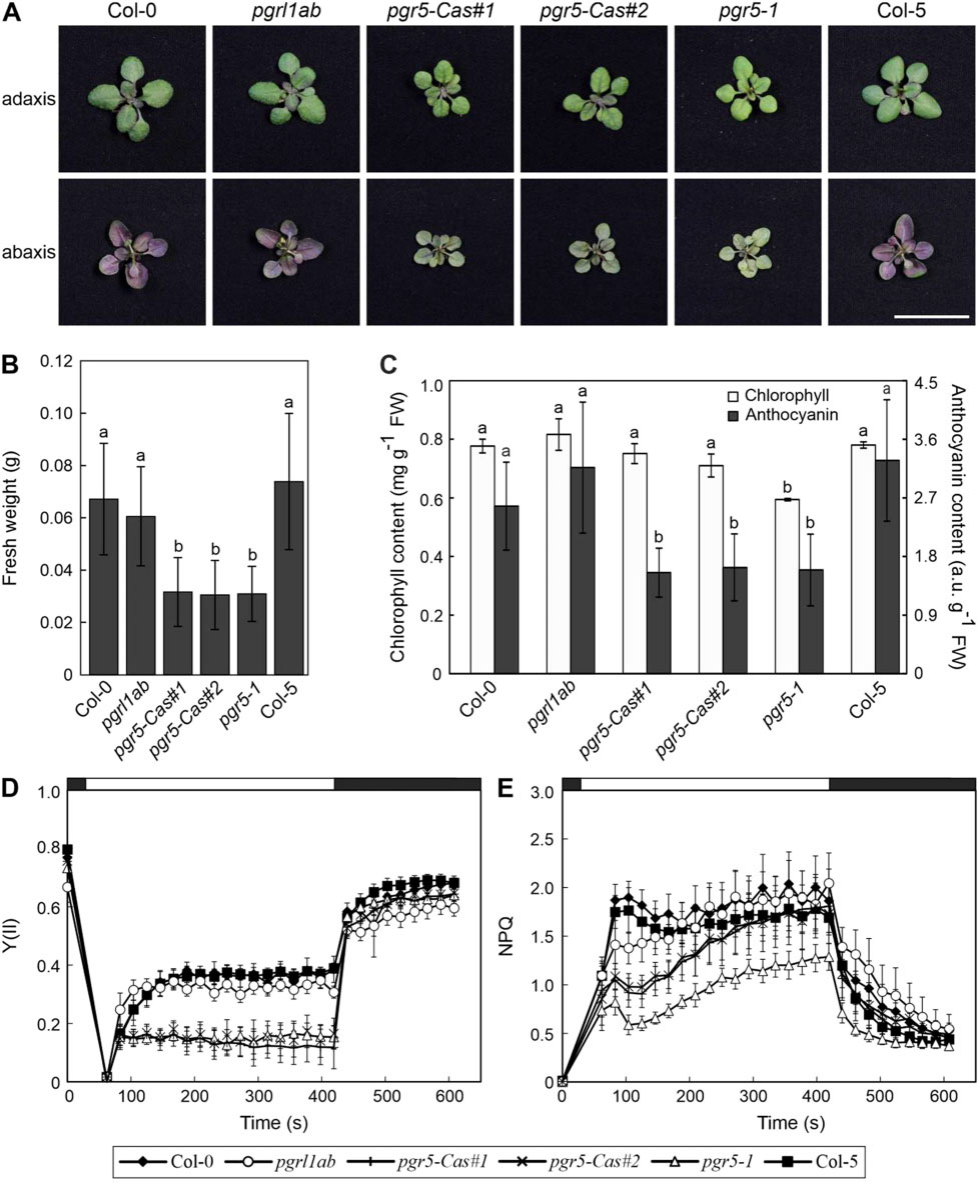
HL phenotype of plants devoid of both PGR5 and PGRL1. A, Growth phenotypes of WT (Col-0 and Col-5) and mutant (*pgrl1ab*, *pgr5-Cas#1*, *pgr5-Cas#2*, and *pgr5-1*) plants grown under HL conditions for 2 weeks: 16-h light/8-h dark, 500 µmol photons m^−2^ s^−1^. The scale bar at the bottom corresponds to 2 cm and for all images in this panel. B, Fresh weight of plants grown as in (A). C, Chlorophyll and anthocyanin contents of plants grown as in (A). Averages of at least three replicates are shown in B and C. Error bars represent standard deviations and different letters on error bars represent statistical differences between the lines (*P* < 0.05) by Tukey’s test. D, Photosystem II quantum yield, Y(II), based on chlorophyll fluorescence monitored in dark adapted plants grown as in (A). Plants were illuminated for 6 min with actinic light (400 μmol photons m^−2^ s^−1^, white bar) and followed by a dark period of 3 min (black bar). Saturating pulses were applied every 20 s. E, NPQ corresponding to the measurements shown in (A). Averages of at least seven plants from two independent experiments are shown. Error bars represent standard deviations.

Plants defective in PGR5-dependent CEF are more sensitive to photo-inhibition (Takahashi et al., 2009; Suorsa et al., 2016; Yamamoto and Shikanai, 2019; Barbato et al., 2020; Rantala et al., 2020). To test whether *pgr5-1* and *pgr5-Cas* also differ in their HL tolerance, we analyzed the growth and pigmentation phenotypes of the different mutants (*pgr5-1*, *pgr5-Cas*, and *pgrl1ab*) under HL conditions (500 µmol photons m^−2^ s^−1^) (Figure 6, A–C). Plants lacking PGR5 but not PGRL1, that is, *pgr5-1* and *pgr5-Cas*, accumulated significantly less fresh weight than the corresponding WT (Figure 6B); moreover, they also accumulated less anthocyanin than WT plants (Figure 6C), resulting in clearly different coloration of plants (Figure 6A). However, in the absence of both PGR5 and PGRL1, that is, in the *pgrl1ab* mutant, growth and anthocyanin content were similar to WT (Figure 6, A–C). Regarding the chlorophyll content, *pgr5-1* was significantly more drastically affected than the other genotypes (Figure 6C), a trend also observed under normal light intensity (Supplemental Table S4). To quantify the effects on photosynthesis in these genotypes under HL conditions, we measured the quantum yield of PSII (Y(II)). In fact, Y(II) was severely impaired in both *pgr5-1* and the *pgr5-Cas* mutants, but not in *pgrl1ab* and WT plants (Figure 6D), providing an explanation for the growth differences between the genotypes. Moreover, the same result regarding Y(II), as well as fresh weight, was observed after growing the plants at milder (280 µmol photons m^−2^ s^−1^) and higher light intensity (800 µmol photons m^−2^ s^−1^), being the differences more pronounced between *pgrl1ab* (similar to WT) and *pgr5-1* and *pgr5-Cas* plants (severely affected), as the light intensity increases (Supplemental Figure S4).

Complementation of the *pgr5-1* mutation with the WT *PGR5* gene also corrected most of the defects seen in the mutant under HL (Supplemental Figures S4 and S7). In particular, we observed that line #4 reached WT levels with respect to fresh weight, anthocyanin content, and Y(II), whereas chlorophyll levels were not restored to WT levels. In the other three *35S::PGR5 pgr5-1* lines, complementation was only partial, most probably owing to the deleterious effects of the markedly enhanced PGR5 levels (Okegawa et al., 2007; Long et al., 2008; Ruhle et al., 2021).

Taken together, these data show that, under HL, the absence of both PGR5 and PGRL1 is less detrimental to photosynthesis and growth than knock-out of PGR5 alone. This implies that, under certain conditions, PGRL1 can become harmful in the absence of PGR5.

## Discussion

### Additional mutations are present in the *pgr5-1* line

Our chloroplast proteome analysis identified 260 *pgr5-1-*specific DEPs (Figure 3 and Supplemental Table S3), including up-regulated proteins involved in the Calvin–Benson cycle and down-regulated components of PSI, PSII, cyt *b*_6_*f*, and the chloroplast ATP synthase, as well as the NDH complex (Table 1 and Supplemental Table S3). The *pgr5-Cas* and *pgrl1ab* mutations had almost no effect on the chloroplast protein content, except PGR5 and/or PGRL1 levels, as expected. Next-generation sequencing of *pgr5-1* revealed the presence of 50 non-synonymous second-site mutations, possibly representing remnants of those generated in the original EMS mutagenesis screen (Shikanai et al., 1999). In addition to *PGR5*, four additional genes coding for chloroplast proteins contained non-silent point mutations, and two of these (PsbO_2_ and HCF136) were found to be downregulated in our proteomic analyses (Tables 1 and 2 and Supplemental Table S3). Indeed, the mutation in PsbO_2_ could have an effect on the *pgr5-1* phenotype, although it has been shown that a second isoform of this protein, PsbO1, is usually the predominant one and PsbO_2_ mutants behave like WT under standard growth conditions (Suorsa et al., 2016). Moreover, the defect in HCF136, an assembly factor of PSII (Meurer et al., 1998), could possibly impair the accumulation of PSII proteins, and might also secondarily affect PSI and chlorophyll levels (Plucken et al., 2002). Therefore, this mutation could in principle explain the exacerbated effect on photosynthesis in *pgr5-1* (relative to *pgr5-Cas*), as well as the lower chlorophyll content and the deficit of proteins such as PsaA or PsaB, but probably not all of the changes observed in the proteomics analysis. However, HCF136 is reduced only to 70% of the WT level in *pgr5-1* (Table 2). Moreover, in principle, we cannot rule out a secondary effect on photosynthesis caused by the other 45 mutated genes that code for non-chloroplast proteins (Supplemental Table S5), or the mutations in the chloroplast proteins CGL20A and ABCI11, which however were not detected in our proteomic analysis (Table 2 and Supplemental Table S3). For instance, plants lacking ABCI11 were reported to show retarded growth and chlorosis (Voith von Voithenberg et al., 2019), while in the absence of CGL20A and CGL20B, which are required for plastid 50S ribosome biogenesis, plants show a pleiotropic phenotype including alterations of the chloroplast proteome, pigment composition, and photosynthetic performance (Reiter et al., 2020). Indeed, introduction of CGL20A into *pgr5-1* can partially complement the mutant phenotype with respect to its pale-green phenotype and altered PSI activity, as shown very recently (Wada et al., 2021). Wada et al. (2021) also identified the *pgr5^hope1^* mutant allele with the same position of mutation in *PGR5* like *pgr5-1*, but with most likely different SNPs, which does not exhibit all phenotypes of *pgr5-1* described in our work, such as the low level of NDH proteins. In addition, the more affected tNPQ induction in the *pgr5-1* mutant compared with *pgrl1ab* or the *pgr5-Cas* lines (Figure 2 and Supplemental Figures S3 and S4) could in principle also be caused by an increased Calvin–Benson cycle capacity. However, although *pgr5-1* accumulates more of Calvin–Benson cycle enzymes (Table 1), it seems not to markedly alter their activity, since the net CO_2_ assimilation in *pgr5-1* is known to be similar or lower than in WT plants (Munekage et al., 2008). Therefore, to clarify what fraction of the changes in the chloroplast proteome and the enhanced impairment of photosynthesis (compared with *pgr5-Cas* and *pgrl1ab*) are attributable to the *pgr5-1* mutation, we complemented the *pgr5-1* mutation with the WT *PGR5* gene (see below).

### PGR5_G130S_ has a negative effect on PGRL1 accumulation and photosynthesis

The complete knock-out of *PGR5* that we generated in this study (*pgr5-Cas*), as well as the *pgrl1ab* mutant (DalCorso et al., 2008) which lacks both PGR5 and PGRL1, display less severe phenotypes with respect to photosynthesis and particularly CEF than does the original *pgr5-1* allele (Munekage et al., 2002), in which very small amounts of the PGR5_G130S_ variant still accumulate (Yamamoto and Shikanai, 2019; Barbato et al., 2020; Figure 1B). A plausible explanation for this is that the remaining PGR5_G130S_ negatively interferes with photosynthesis. Indeed, it has been shown that PGRL1 cannot productively interact with PGR5_G130S_ in the heterologous *Synechocystis* system (Dann and Leister, 2019) and, under conditions in which the PGR5_G130S_ protein can accumulate to moderate amounts (in the *pgr5-1 pgrl2-1* mutant), the mutated PGR5 protein fails to support CEF and does not restore seedling viability under FL (Ruhle et al., 2021). However, the large number of second-site mutations in the *pgr5-1* line, including four mutations in genes for chloroplast proteins (see above), made it necessary to complement the *pgr5-1* mutation with the WT *PGR5* gene to clarify the impact of *pgr5-1* on the observed photosynthetic phenotype. In this case, remaining differences between the complemented lines and the original WT can in principle be attributed to the second-site mutations rather than to the *pgr5-1* mutation. Indeed, some *pgr5-1-*specific changes in the abundance of chloroplast proteins could not be abrogated by the expression of WT PGR5 (Figure 4C), implying that these changes are indeed caused by second-site mutations. Moreover, expression of the WT PGR5 protein in the *pgr5-1* background almost restored WT-like photosynthesis (Figure 5 and Supplemental Figure S5), and therefore the PGR5_G130S_ protein must be responsible for most of the enhanced photosynthetic defect observed in *pgr5-1*. But how can the mutated PGR5 be more harmful than having no PGR5 at all? “Free” PGR5 without channeling of its activity by PGRL1 has been shown to be detrimental to photosynthesis, but the PGR5_G130S_ variant lacks CEF activity (Ruhle et al., 2021). Nevertheless, PGR5_G130S_ might still be able to negatively interact with other proteins. In fact, the PGRL1 protein was less abundant in *pgr5-1* than in *pgr5-Cas* and a second PGRL1 band clearly appears only in the *pgr5-1* background (Figure 1B), which could be a degradation product or a post-translationally modified form of PGRL1 induced by PGR5_G130S_. Complementation of *pgr5-1* with WT PGR5 restored PGRL1 levels, but had no effect on the putative degradation product of PGRL1 (Figure 4B). Hence, our results imply that PGR5_G130S_ destabilizes PGRL1 although the precise origin of the second PGRL1 signal in Western blots remains elusive. However, destabilization of PGRL1 alone cannot explain the enhanced photosynthetic defect in *pgr5-1* compared with *pgrl1ab*, since the latter genotype lacks both PGR5 and PGRL1 and still is more similar to *pgr5-Cas* than to *pgr5-1* (Figure 2 and Supplemental Figure S3). Therefore, PGR5_G130S_ might also negatively interact with other proteins, and indeed the markedly altered chloroplast protein profile in *prg5-1* provides ample candidates for such interacting partners.

It should be noted here that complementation of the *pgr5-1* mutation with the WT *PGR5* gene under control of the 35S promoter rather than the original promoter might itself lead to phenotypic artifacts due to altered levels, timing, and localization of PGR5 expression compared with the WT. Nevertheless, the use of a set of four lines with a range of PGR5 expression levels allowed us to monitor the effects of different levels of PGR5 expression, and the main aim of the experiment—to test whether WT PGR5 can abrogate the enhanced effects of the *pgr5-1* mutation on photosynthesis—was achieved by using this approach.

### “Free” PGRL1 can have harmful effects under certain conditions

It is known that plants deficient in PGR5-dependent CEF are more sensitive to photo-inhibition (Takahashi et al., 2009; Suorsa et al., 2016; Yamamoto and Shikanai, 2019; Barbato et al., 2020; Rantala et al., 2020). Indeed, when we grew *pgr5-1* and *pgr5-Cas* mutants under HL they were impaired in growth, photosynthetic performance, and anthocyanin production (Figure 6 and Supplemental Figure S4). Interestingly, a decrease in anthocyanin levels has also been observed in plants that overexpress PGR5 after exposure to HL, which was attributed to a defect in sensing the light intensity (Long et al., 2008). However, in our experiment, *pgrl1ab* behaved like WT under HL intensities with respect to its growth phenotype and Y(II) (Figure 6 and Supplemental Figure S4). In consequence, in the absence of PGR5, PGRL1 itself is detrimental under HL conditions. This, together with the recent observation that “free” PGR5, that is, in the absence of both PGRL1 and PGRL2, has a negative effect on the photosynthetic performance of the plant under low-light conditions (Ruhle et al., 2021), points to distinct functions of PGR5 and PGRL1 under different light conditions.

## Conclusions

With the *pgr5-Cas* line, a genetic resource is now available that allows one to study photosynthesis without PGR5 and without the side effects present in the original *pgr5-1* line due to second-site mutations and the additional negative effect of the amino-acid substitution in PGR5. In fact, although PGR5_G130S_ only accumulates in tiny amounts under normal conditions, it appears to have a more deleterious impact on CEF than does the complete loss of PGR5.

The current view is that PGR5 is central to CEF and can function without PGRL1—although it has negative effects in the absence of the latter (Ruhle et al., 2021). Here, we show that the reverse is also true: in the absence of PRG5, PGRL1 becomes deleterious under HL. We therefore speculate that in this case it might direct its original channeling function to other proteins—with detrimental effects. Future studies will be needed to clarify how this rogue activity of PGRL1 affects growth and anthocyanin production under HL.

## Materials and methods

### Plant material

In this study, the Arabidopsis (*A. thaliana*) ecotypes Col-0 and Col-5 were used as WT controls. The Arabidopsis *pgr5-1* (G130S point mutation) and *pgrl1ab* mutants were previously described (Munekage et al., 2002; DalCorso et al., 2008, respectively). To generate the *pgr5-Cas* line, the pHEE401-E vector was used, which has an egg-cell-specific promoter (Wang et al., 2015). The specific guide RNA (gRNA) was designed using the web tool CRISPOR (Concordet and Haeussler, 2018) and cloned into pHEE401-E as described (Wang et al., 2015). Col-0 plants were transformed with the construct using *Agrobacterium tumefaciens* GV3101 (Clough and Bent, 1998). Positive transformants were selected in the first generation (T1) on plates containing Murashige and Skoog (MS) salt medium (1×), 25 μg mL^−1^ hygromycin, 1% (w/v) plant agar, and 1% (w/v) sucrose. The *PGR5* gene was sequenced in the surviving plants to select for homozygous *pgr5* mutants. The *pgr5-1* lines overexpressing the Arabidopsis PGR5 protein (*35S*::*PGR5 pgr5-1* #1-4) were kindly provided by Prof. Toshiharu Shikanai and Dr. Hiroshi Yamamoto. To generate these lines, the genomic DNA fragment containing *PGR5* was amplified by PCR and cloned between HindIII and SacI sites of the binary vector pBI121. The *pgr5-1* mutant was transformed by floral dipping using *A. tumefaciens* GV3101 (Clough and Bent, 1998) and transformed plants were selected on 0.5-strength MS-agar plates containing kanamycin. Primers used for gRNA, sequencing, and cloning are listed in Supplemental Table S8.

### Plant growth conditions

Seeds from mutant and WT plants were sown on potting soil and stratified for 3 days at 4°C and grown in climate chambers under various light intensities and day lengths (LD, 16-h light [100 µmol photons m^−2^ s^−1^]/8-h darkness; SD, 8-h light [100 µmol photons m^−2^ s^−1^]/16-h darkness; HL, 16-h light [500 µmol photons m^−2^ s^−1^]/8-h darkness; FL, 12-h light/12-h darkness, with cycles of 5 min at 50 μmol photons m^−2^ s^−1^ and 1 min at 500 μmol photons m^−2^ s^−1^ during the light period). Temperature (22°C/20°C during the day/night cycle) and relative humidity (60%) were strictly controlled under all conditions. Fertilizer was added according to the manufacturer’s recommendations (Osmocote Plus; Scotts, Nordhorn, Germany).

### Chlorophyll fluorescence and P700 measurements

Photosynthetic performance was monitored in vivo by simultaneously measuring chlorophyll *a* fluorescence and P700 absorbance changes using a Dual/KLAS-NIR spectrophotometer (Walz, Effeltrich, Germany). Induction-recovery curves (IRCs) were constructed based on data obtained from attached leaves of plants that had been dark-adapted for 30 min and treated as follows. Blue actinic light (110 μmol photons m^−2^ s^−1^) was applied for 6 min, followed by 3 min of darkness. Saturating pulses of 8,000 μmol photons m^−2^ s^−1^ and 0.3 s duration were applied every 20 s to determine photosynthetic parameters, which were calculated by the DUAL/KLASNIR software using the previously described equations (Klughammer and Schreiber, 2008a, 2008b, 2016). CEF was examined by analyzing the transient rise in NPQ after the dark-to-light transition (Munekage et al., 2002; DalCorso et al., 2008; Ruhle et al., 2021) and the maximum value reached was denoted as tNPQ_max_. Light curves were obtained by applying stepwise increasing actinic light intensities every 3 min, and saturation pulses were applied at the end of each step to calculate photosynthetic parameters as described for IRC. Chlorophyll *a* fluorescence of plants grown under HL conditions was monitored following the same IRC protocol, but applying 450 μmol photons m^−2^ s^−1^ of blue actinic light and using an Imaging-PAM spectrophotometer (Walz).

To monitor the fast oxidation kinetics of P700, the Dual-PAM-100 spectrophotometer (Walz) was used. Dark-adapted leaves (30 min) were exposed to 5 s of actinic light (600 μmol photons m^−2^ s^−1^) followed by 2 s of darkness, to induce maximum reduction of P700. The oxidation of P700 was recorded over 23 s of far-red (FR) light as the difference between the transmittance signals at 830 and 875 nm. Curves were normalized by setting the minimum absorbance after FR illumination to 0 and the maximum to 1 to determine the oxidation half time of P700^+^, denoted as t_0.5_P700ox.

### Protein extraction and immunoblot analysis

For quantification of PGR5 and PGRL1, rosette leaves (about 50 mg fresh weight) from 3-week-old plants grown under LD conditions were ground with liquid nitrogen and homogenized in 500 µL of 2× Tricine buffer containing 8% [w/v] SDS, 24% [w/v] glycerol, 15 mM DTT, and 100 mM Tris/HCl pH 6.8. The homogenate was incubated for 5 min at 70°C and centrifuged for 10 min at 13,000 × *g*. Solubilized leaf proteins corresponding to 1 mg (for PGRL1 detection) and 3 mg (for PGR5 detection) fresh weight were loaded onto 10% Tricine-SDS PA gels and subjected to electrophoresis (Schagger, 2006).

Chloroplast proteins were isolated as previously described (Sun et al., 2011). Briefly, leaf samples from dark-adapted plants were homogenized in buffer containing 330 mM sorbitol, 20 mM Tricine-NaOH (pH 7.6), 10 mM Na_2_CO_3_, 5 mM EGTA (ethylene glycol-bis(β-aminoethyl ether)-N,N,N′,N′-tetraacetic acid), 0.1% [w/v] BSA (bovine serum albumin), and 330 mg l-ascorbate. After filtration through two layers of Miracloth, the homogenates were centrifuged for 5 min at 2,000 × *g*. Crude chloroplasts were resuspended in buffer containing 330 mM sorbitol, 20 mM HEPES-KOH (pH 7.6), 10 mM Na_2_CO_3_, 5 mM MgCl_2_, 2.5 mM EDTA (ethylenediamine tetraacetic acid), and 0.1% [w/v] BSA, and layered on a discontinuous (40%/70% [v/v]) Percoll gradient. Intact chloroplasts were isolated from the interface after centrifugation for 15 min at 1,500 × *g* and ruptured by hypotonic shock on ice for 10 min. Protein concentrations were determined using the ROTI-Quant (Carl Roth, Karlsruhe, Germany) protein assay according to the manufacturer‘s protocol. Aliquots (10 μg) of total chloroplast proteins were solubilized in SDS loading buffer (50 mM Tris–HCl, pH 6.8, 6% [v/v] glycerol, 2% [w/v] SDS, 1% [v/v] 2-mercaptoethanol, and 0.02% [w/v] bromophenol blue) and fractionated by SDS-PAGE (12% polyacrylamide).

The resolved proteins were transferred to polyvinylidene fluoride (PVDF) membranes (Immobilon-P; Millipore, Burlington, MA, USA) using the Trans-Blot Turbo system (Bio-Rad, Hercules, CA, USA) or capillary transfer (Dann and Leister, 2019) in the case of PGR5 detection. PVDF membranes were blocked with 5% [w/v] milk in TBS-T (10 mM Tris, pH 8.0, 150 mM NaCl, and 0.1% Tween 20) and probed with antibodies against PGR5 (1/2,500 dilution; provided by Prof. Shikanai) and PGRL1 (1/10,000; DalCorso et al., 2008). Primary antibodies directed against FBPase (1/50,000; AS19 4319), NdhB (1/1,000; AS16 4064), PsaA (1/5,000; AS06 172), and PsbO (1/5,000; AS06 142-33) were obtained from Agrisera (Vännäs, Sweden). Equal loading was verified by staining PVDF membranes with Coomassie brilliant blue R-250 dye. Signals were visualized with enhanced chemiluminescence using the Pierce ECL Western Blotting substrate reagent (ThermoFisher Scientific, Waltham, MA, USA) and an ECL reader system (Fusion FX7; VWR, Radnor, PA, USA). Signals were quantified with ImageJ software (National Institutes of Health).

### Proteome analysis

Proteome profiling was conducted using protein extracts of three independent chloroplast extractions. Proteome preparation, trypsin digestion, and liquid chromatography-tandem mass spectrometry (LC‐MS/MS) were performed as described previously (Marino et al., 2019).

Raw files were processed using the MaxQuant software 1.6.17.0 (Cox and Mann, 2008). Peak lists were searched against the Arabidopsis reference proteome (Uniprot, www.uniprot.org, version April 2021) using the built‐in Andromeda search engine (Cox et al., 2011) with default settings. Proteins were quantified across samples using the label‐free quantification algorithm (Cox et al., 2014) and the match-between-runs option was enabled. Downstream statistical analysis was performed using Perseus version 1.6.15.0 (Tyanova et al., 2016) and RStudio version 1.2.5019 (Team, 2019). Significantly differentially abundant protein groups were calculated employing the R/Bioconductor package limma (Ritchie et al., 2015), with *P*-values adjusted for multiple comparisons according to the Benjamini–Hochberg approach (Benjamini and Hochberg, 1995). Proteins with a fold change relative to the WT larger than 1.5 (up-regulated) or lower than 0.66 (down-regulated) and with an FDR < 0.05 were considered to show a significant change.

The mass spectrometry proteomics data have been deposited to the ProteomeXchange Consortium via the PRIDE (Perez-Riverol et al., 2019) partner repository with the dataset identifier PXD028563.

### Whole-genome resequencing and data analysis

For DNA isolation, leaves from a pool of 50–60 plants were ground in liquid nitrogen and incubated in lysis buffer (0.4 M sucrose, 10 mM Tris–HCl pH 7.0, 1% [v/v] β-ME, and 1% [v/v] Triton) on ice for 15 min. The lysate was then filtered and centrifuged for 15 min at 1,200 × *g* and 4°C, and the pellet was resuspended in 1 mL of lysis buffer and centrifuged again for 15 min (600 × *g*, 4°C). DNA was isolated from the supernatant using the DNeasy Plant Mini Kit (Qiagen, Hilden, Germany). Two micrograms of DNA was used to prepare 350-bp-insert libraries for 150-bp paired-end sequencing (Novogene Biotech, Beijing, China) on an Illumina HiSeq 2500 system (Illumina, San Diego, USA) with standard Illumina protocols. The sequencing depth was at least 7 G raw data per sample, which corresponds to higher than 50-fold coverage of the Arabidopsis genome. After grooming of FASTQ files, adaptors were removed with Trimmomatic (Bolger et al., 2014), reads were mapped with BWA (Li and Durbin, 2009) to the TAIR10 annotation with the parameters “mem -t 4 -k 32 –M,” and duplicates were removed by SAMtools (Li et al., 2009) with the rmdup tool. SNPs were identified using SAMtools (Li et al., 2009) with the following parameters: “mpileup -m 2 -F 0.002 -d 1000.” Only SNPs that were supported by more than 8 reads, and whose mapping quality was >20, were retained. To identify the SNPs specific for *pgr5-1*, the SNPs between *pgr5-1*, Col-5, and Col-0 were compared. The resulting *pgr5-1*-specific SNP list was subjected to the web application CandiSNP (Etherington et al., 2014), which generates SNP density plots. The output list of CandiSNP was screened for non-synonymous amino-acid changes and the G/C-to-A/T transitions that were likely to be caused by EMS, with a special focus on the chromosome with the highest density of SNPs with an allele frequency of >0.75.

### Pigment measurements

For chlorophyll measurements, about 100 mg of ground leaves were weighed and incubated in 2 mL of 80% [v/v] acetone overnight at 4°C in the dark and shaking. After extraction, chlorophyll levels were measured spectrophotometrically as described (Lichtenthaler and Wellburn, 1983) and normalized to fresh weight. The anthocyanin content was measured as described (Xu et al., 2019). Aliquots of ground rosettes (about 100 mg) from different plants were agitated in 300 µL of 1% [v/v] HCl in methanol overnight at 4°C in the dark. Then 200 µL of distilled water and 500 µL of chloroform were added, the mixture was vortexed, and briefly centrifuged to separate anthocyanins from chlorophylls. The total anthocyanin content was determined as described (Neff and Chory, 1998), by measuring A530 and A657 of the aqueous phase using a spectrophotometer (Ultrospec 2100 *pro*; Biochrom, Holliston, MA, USA). The equation (A530 − 0.25 × A657) × TV/FW was used to quantify the relative amount of anthocyanin, where TV = total volume of the extract (in milliliters) and FW = fresh weight of starting material (in grams).

### Statistical analyses

Statistically significant differences, except for the proteome analysis, were tested by applying one-way ANOVA with post hoc Tukey HSD test (https://astatsa.com/, version August 2021).

## Accession numbers

Sequence data from this article can be found in the GenBank/EMBL data libraries under accession numbers: At2g05620 (*PGR5*), At4g22890 (*PGRL1A*), and At4g11960 (*PGRL1B*).

## Supplemental data

The following materials are available in the online version of this article.

**Supplemental Figure S1.** Protein content in the different genotypes.

**Supplemental Figure S2.** Photosynthetic performance and assessment of proton motive force in the different genotypes.

**Supplemental Figure S3.** Seedling growth and photosynthetic phenotypes.

**Supplemental Figure S4.** Growth and photosynthetic performance of the different genotypes under multiple irradiances.

**Supplemental Figure S5.** Photosynthetic performance of *pgr5-Cas* lines compared with *pgr5-1* and *pgrl1ab* mutants under different irradiances.

**Supplemental Figure S6.** tNPQ at different light intensities.

**Supplemental Figure S7.** Complementation of the *pgr5-1* mutant under HL conditions.

**Supplemental Table S1.** IRC raw data for *pgr5* deficient mutants.

**Supplemental Table S2.** P700ox raw data for *pgr5* deficient mutants.

**Supplemental Table S3.** List of all proteins identified by shotgun proteomic and GO analysis.

**Supplemental Table S4.** Chlorophyll contents of WT (Col-0 and Col-5) and mutant (*pgrl1ab*, *pgr5-Cas#1*, *pgr5-Cas#2*, and *pgr5-1*) plants grown under LD and SD conditions for 3 and 6 weeks, respectively.

**Supplemental Table S5.** List of SNPs found in *pgr5-1* but not in Col-5 or Col-0.

**Supplemental Table S6.** IRC raw data for *pgr5-1* complemented lines.

**Supplemental Table S7.** P700ox raw data for *pgr5-1* complemented lines.

**Supplemental Table S8.** Oligonucleotide sequences used for gRNA, sequencing, and cloning.

**Supplemental Methods.** Supplemental Materials and Methods.

## Supplementary Material

kiac362_Supplementary_Data

## Notes

D.L. and B.N. designed the research and wrote the manuscript with the support of G.M. and J.-F.P. J.-F.P. generated the pgr5-Cas lines and performed the experiments. G.M. conducted the proteomic analysis. B.R. performed the ECS measurements. T.K. analyzed the resequencing data. D.L. and B.N. All authors have read and approved the final manuscript.

The author responsible for distribution of materials integral to the findings presented in this article in accordance with the policy described in the Instructions for Authors (https://academic.oup.com/plphys/pages/general-instructions) is: Dario Leister (leister@lmu.de).
