# Supplementary material for: Commonalities and specialties in photosynthetic functions of PROTON GRADIENT REGULATION5 variants in Arabidopsis
Source: Plant Physiol. 2022 Aug 10;190(3):1866–82. doi: 10.1093/plphys/kiac362 (PMC9614465; doi:10.1093/plphys/kiac362)
Supplement: kiac362_Supplementary_Data [file kiac362_supplementary_data.zip › Supplemental Materials_FINAL.pdf]

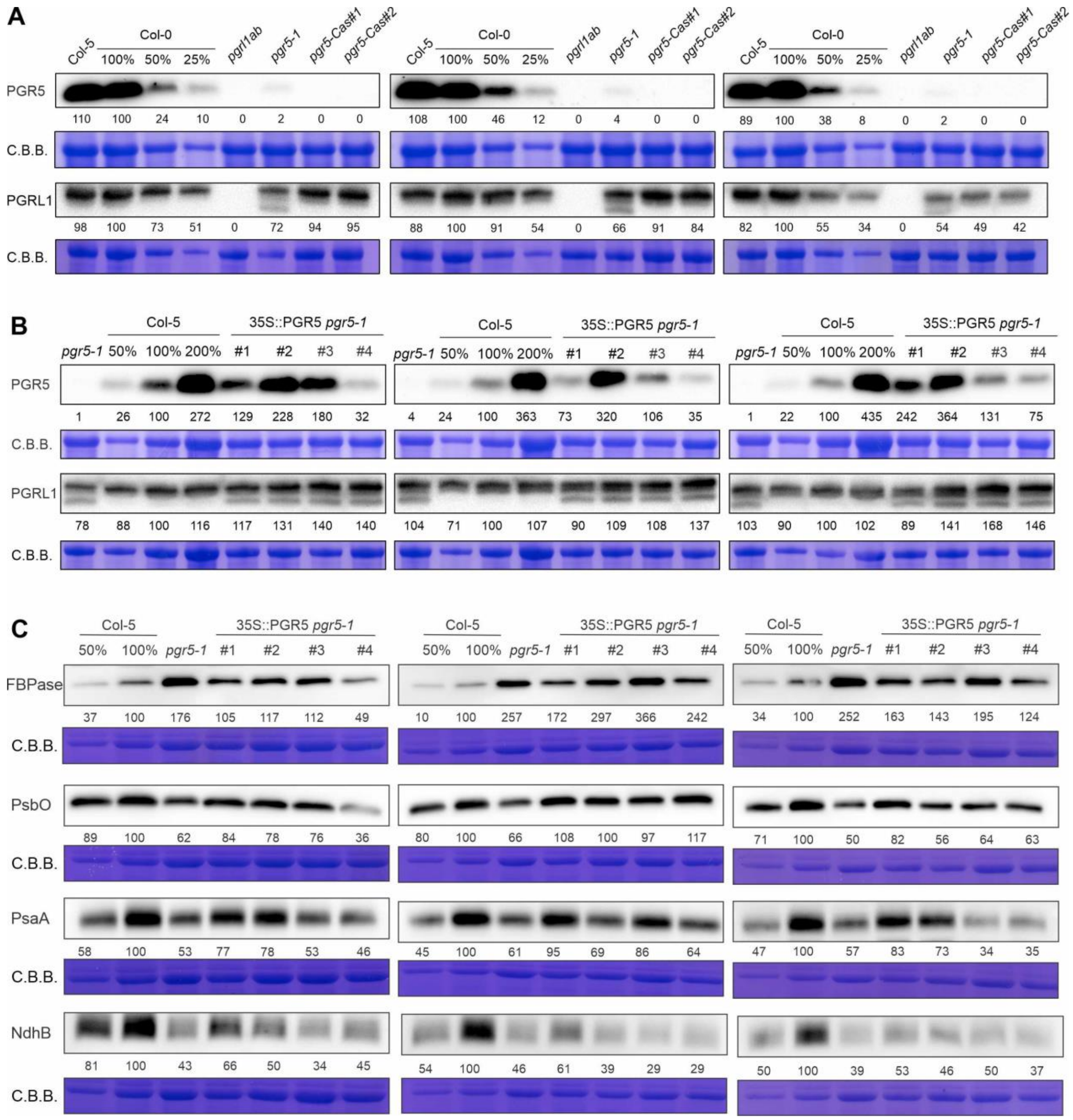

Supplemental Figure S1. Protein content in the different genotypes. **A**, Aliquots of total leaf proteins were isolated from Col-5, Col-0, *pgrl1ab*, *pgr5-1*, *pgr5-Cas#1* and *pgr5-Cas#2* plants after 3 weeks of growth under long-day conditions, fractionated by SDS-PAGE under reducing conditions, and subjected to immunoblotting using PGR5- or PGRL1-specific antibodies. Decreasing amounts of Col-0 were loaded as a control. Protein samples were adjusted according to fresh weight. PVDF membranes were stained with Coomassie brilliant blue (C.B.B.) to show protein loading. The gels of the second replicate (center) are the same images as shown in Figure 1B. **B**, Aliquots of total leaf proteins from Col-5, *pgr5-1* and *35S::PGR5 pgr5-1* (#1-4) plants grown for 3 weeks under long-day conditions were fractionated by SDS-PAGE and subjected to immunoblotting as described in (**A**). Varying amounts of Col-5 were

loaded as indicated. The gels from the first replicate (left) are the same images as shown in Figure 4B. **C**, Immunoblot analysis of representative chloroplast proteins. Chloroplasts were isolated from Col-5, *pgr5-1* and *35S::PGR5 pgr5-1* (#1-4) plants after 7 weeks of growth under short-day conditions. Protein samples were fractionated by SDS-PAGE and subjected to immunoblotting using the indicated antibodies specific for proteins of the Calvin-Benson cycle: (FBPase), PSI (PsaA), PSII (PsbO) and the NDH complex (NdhB). Decreasing amounts of Col-5 were loaded. PVDF membranes were stained with Coomassie brilliant blue (C.B.B.) as loading control. The gels from the first replicate (left) are the same images as shown in Figure 4C. In all instances, three independent replicates are presented as well as the values corresponding to the quantification of the intensity of each band relative to Col-0 or Col-5 (100%). Intensities of bands were quantified using ImageJ software (National Institutes of Health).

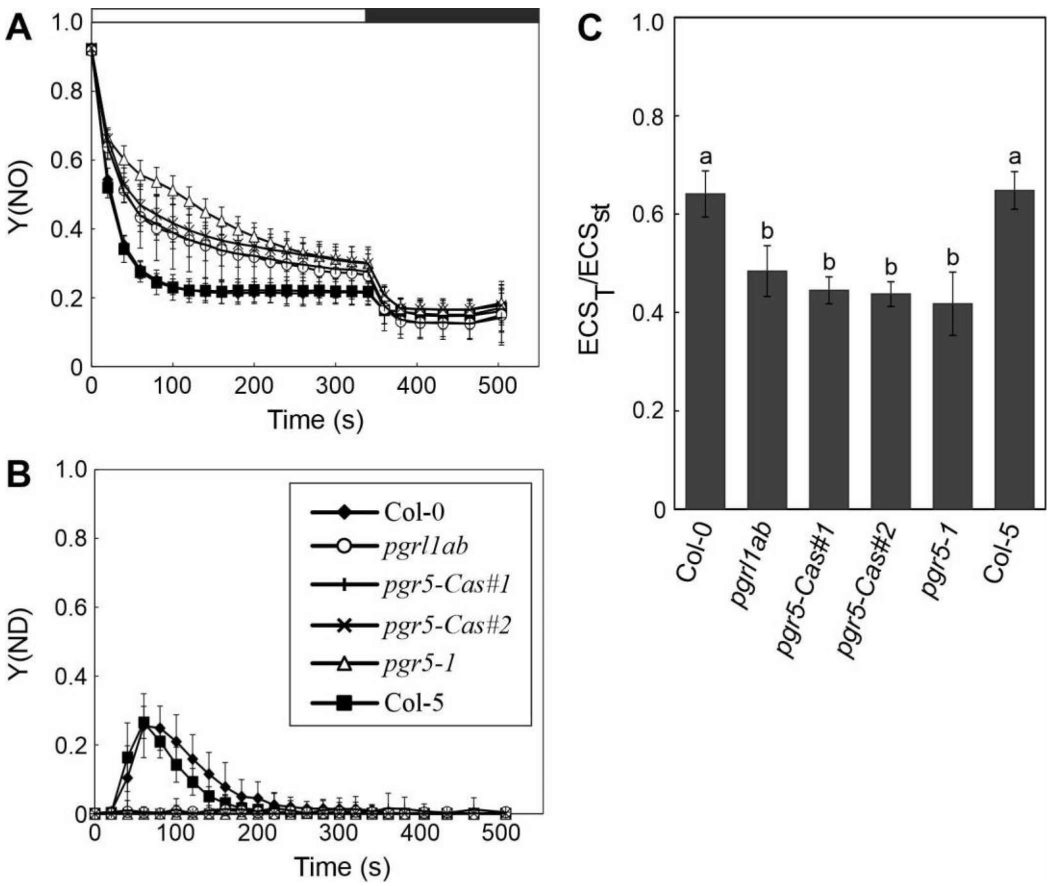

Supplemental Figure S2. Photosynthetic performance and assessment of proton motive force in the different genotypes. **A**, Quantum-yield of non-regulated energy dissipation (Y(NO)) was determined during the measurements shown in **Figure 2A**. **B**, PSI donor-side (Y(ND)) imitation was determined during the measurements shown in **Figure 2B**. Averages of at least 8 replicates from two independent experiments are shown. Error bars represent standard deviations (SD). The white bar indicates the illumination period of 6 min with actinic light (100 µmol photons m^{-2} s^{-1}) and the black bar indicates the dark period of 3 min. **C**, Electrochromic shift (ECS) was measured to assess the proton motive force (pmf) in Col-0, *pgrl1ab*, *pgr5-Cas#1*, *pgr5-Cas#2*, *pgr5-1* and Col-5 plants after 5 weeks of growth under a 12 h light / 12 h dark photoperiod. Plants were exposed to 340 µmol photons m^{-2} s^{-1} of actinic light for 15 min, and the change in absorbance at 515 nm (ECS_T) was recorded in dark-interval relaxation kinetics. ECS_T values were normalized to the change in absorbance at 515 nm induced by a single turnover flash prior to exposure to actinic light (ECSst). Data represent means ± SD ($n \geq 7$). Different letters above error bars represent statistical difference ($p < 0.05$) as determined by Tukey's test.

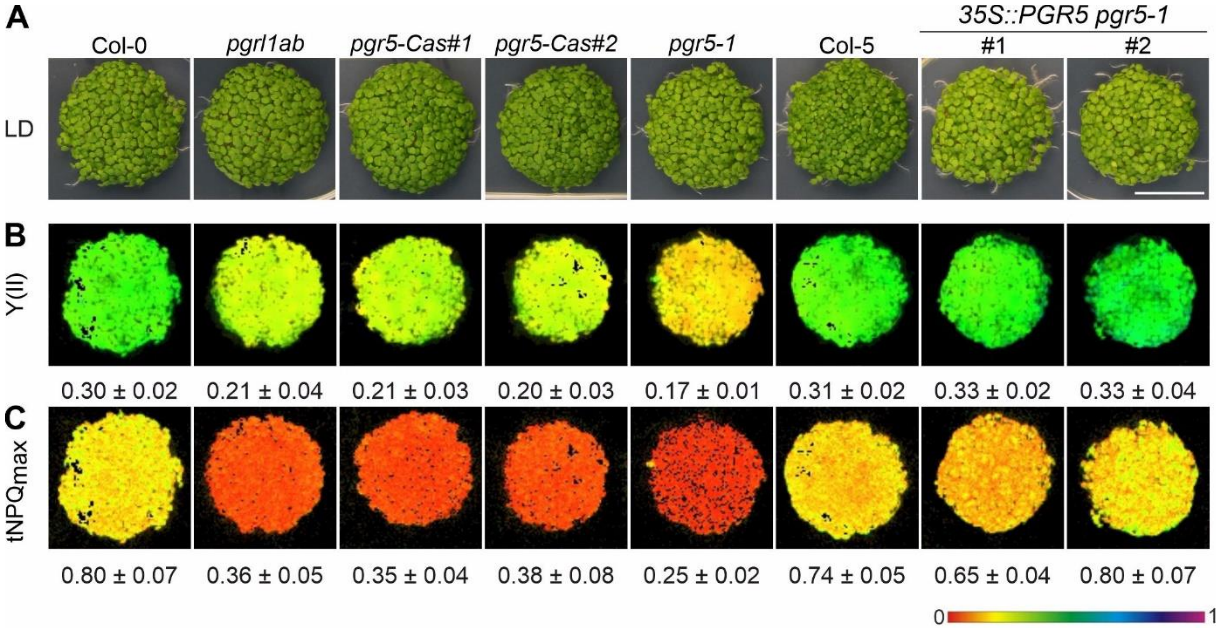

Supplemental Figure S3. Seedling growth and photosynthetic phenotypes. **A**, One-week old plants [WT (Col-0 and Col-5), *pgrl1ab*, *pgr5-Cas#1*, *pgr5-Cas#2*, *pgr5-1* and two different *pgr5-1* lines overexpressing PGR5 (*35S::PGR5 pgr5-1* #1 and #2)] grown under long-day (LD) conditions in ½ MS plates. **B**, PSII quantum yield (Y(II)) of dark-adapted plants grown as in (**A**) after 5 minutes of illumination with 110 µmol photons m^{-2} s^{-1} of actinic light, using the ImagePAM fluorimeter. **C**, Non-photochemical quenching (NPQ) was determined during the measurements in (**B**) and the maximal transient NPQ ($tNPQ_{max}$) obtained in the first seconds after illumination is shown. Averages of at least 6 replicates ± standard deviations are indicated; signal intensities correspond to the colour scale at the bottom of the panel. The scale bar at the bottom of (A) corresponds to 1 cm for all images in this panel.

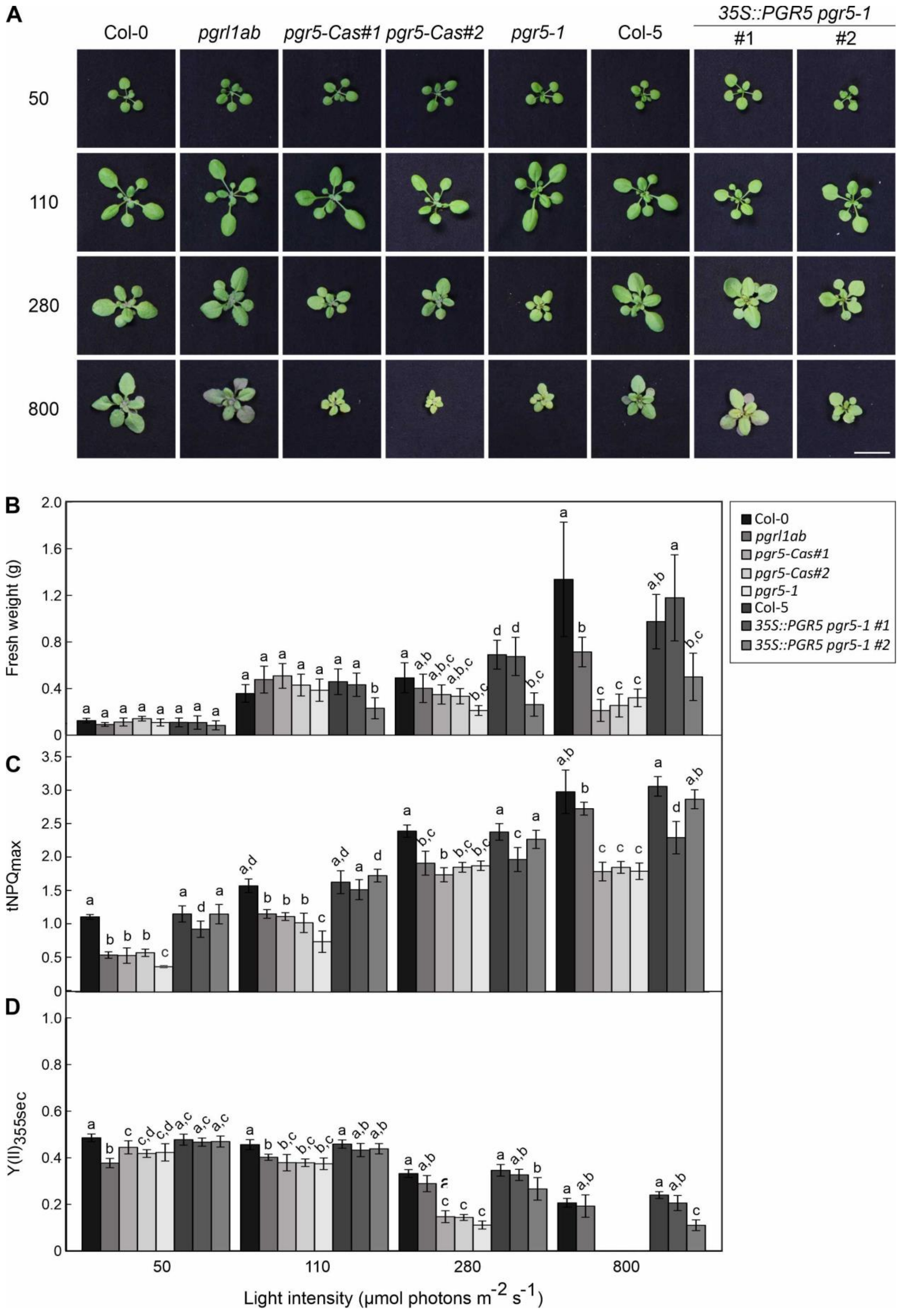

Supplemental Figure S4. Growth and photosynthetic performance of the different genotypes under multiple irradiances. **A**, Growth phenotypes of plants [wild-type (Col-0 and Col-5), mutants (*pgrl1ab*,

pgr5-Cas#1, pgr5-Cas#2 and *pgr5-1*), and two different *pgr5-1* lines overexpressing PGR5 (*35S::PGR5 pgr5-1,* #1 and #2)] grown for 2 weeks under different light intensities: 50, 110, 280 and 800 µmol photons m^{-2} s^{-1}. **B**, Fresh weight of plants grown as in (**A**). **C**, Average-maximal transient NPQ ($tNPQ_{max}$) in the first seconds after a dark to light transition (same actinic light intensity than growth) in dark-adapted plants grown as in (**A**). **D**, Photosystem II quantum yield, Y(II), in dark-adapted plants grown as in (**A**) after 5 minutes of illumination with actinic light at the same intensity than the light growth condition (steady-state). Averages of at least six replicates are shown. Error bars represent standard deviations and different letters on error bars represent statistical difference between the lines in each light condition ($p < 0.05$) by Tukey's test. The scale bar at the bottom corresponds to 2 cm for all images in this panel.

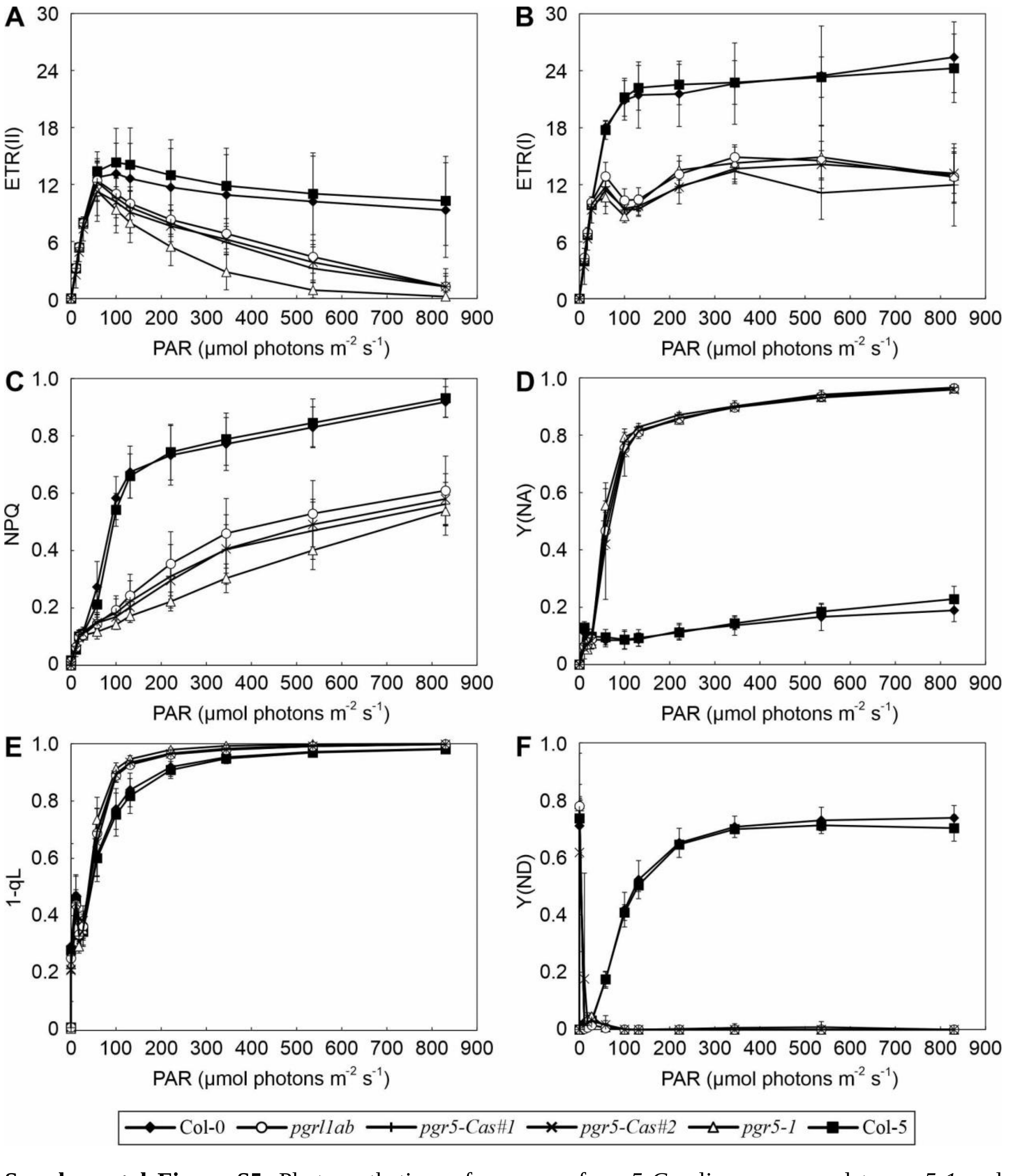

Supplemental Figure S5. Photosynthetic performance of *pgr5-Cas* lines compared to *pgr5-1* and *pgrl1ab* mutants under different irradiances. **A**, The PSII electron transport rate (ETR(II)), based on chlorophyll fluorescence, was determined in dark-adapted plants (long-day, 3 weeks old) after 3 min of stepwise increasing photosynthetically active radiation (PAR). Saturation pulses were applied at the end of each illumination period. **B**, PSI I electron transport rate (ETR(I)), based on absorbance measurements at 830 nm and 875 nm, was determined in the same plants as in (**A**) and following the same light-curve and saturation pulse analysis. **C** and **E**, Non-photochemical quenching (NPQ) and

reduction state of the plastoquinone pool determined as 1-qL obtained from the measurements underlying (**A**). **D** and F, PSI acceptor-side (Y(NA)) and donor-side (Y(ND)) limitations obtained from the same measurements underlying (**B**). Averages of five replicates are shown. Error bars represent standard deviations.

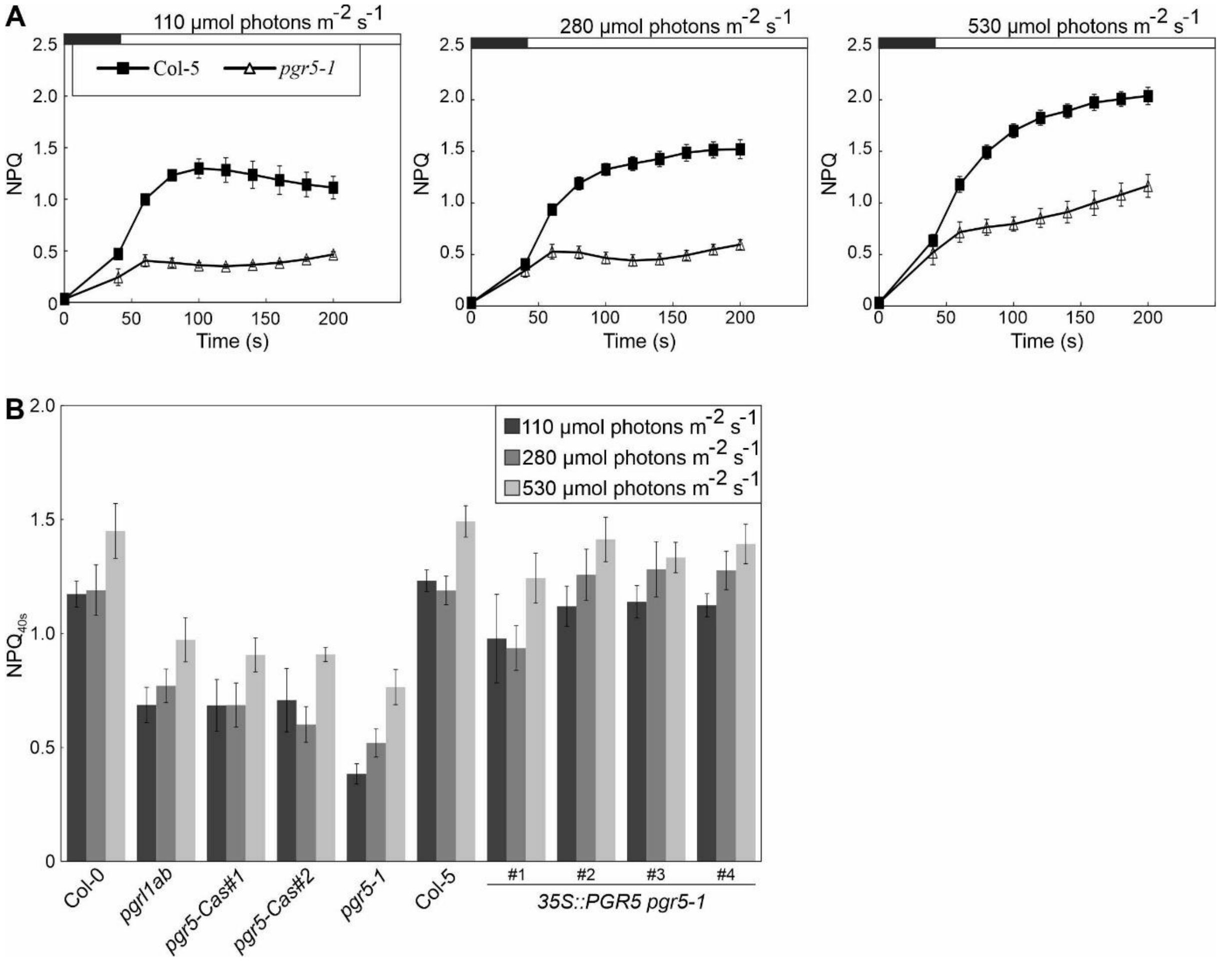

Supplemental Figure S6. Transient NPQ at different light intensities. **A**, NPQ induction based on chlorophyll fluorescence monitored in dark adapted plants (Col-5 and *pgr5-1*) grown for 3 weeks under LD conditions (100 µmol photons m^{-2} s^{-1}). Plants were illuminated with actinic light of different intensities (110, 280 and 530 µmol photons m^{-2} s^{-1}) during 200 s and saturating pulses were applied every 20 s. **B**, NPQ values from measurements as in (A), after 40 seconds of illumination (NPQ_{40s}) with each light intensity and for each genotype [WT (Col-0 and Col-5), *pgrl1ab*, *pgr5-Cas#1*, *pgr5-Cas#2*, *pgr5-1* and four different *pgr5-1* lines overexpressing PGR5 (*35S::PGR5 pgr5-1*, #1-4)] grown as in (A). Average of 8 replicates ± standard deviations are represented.

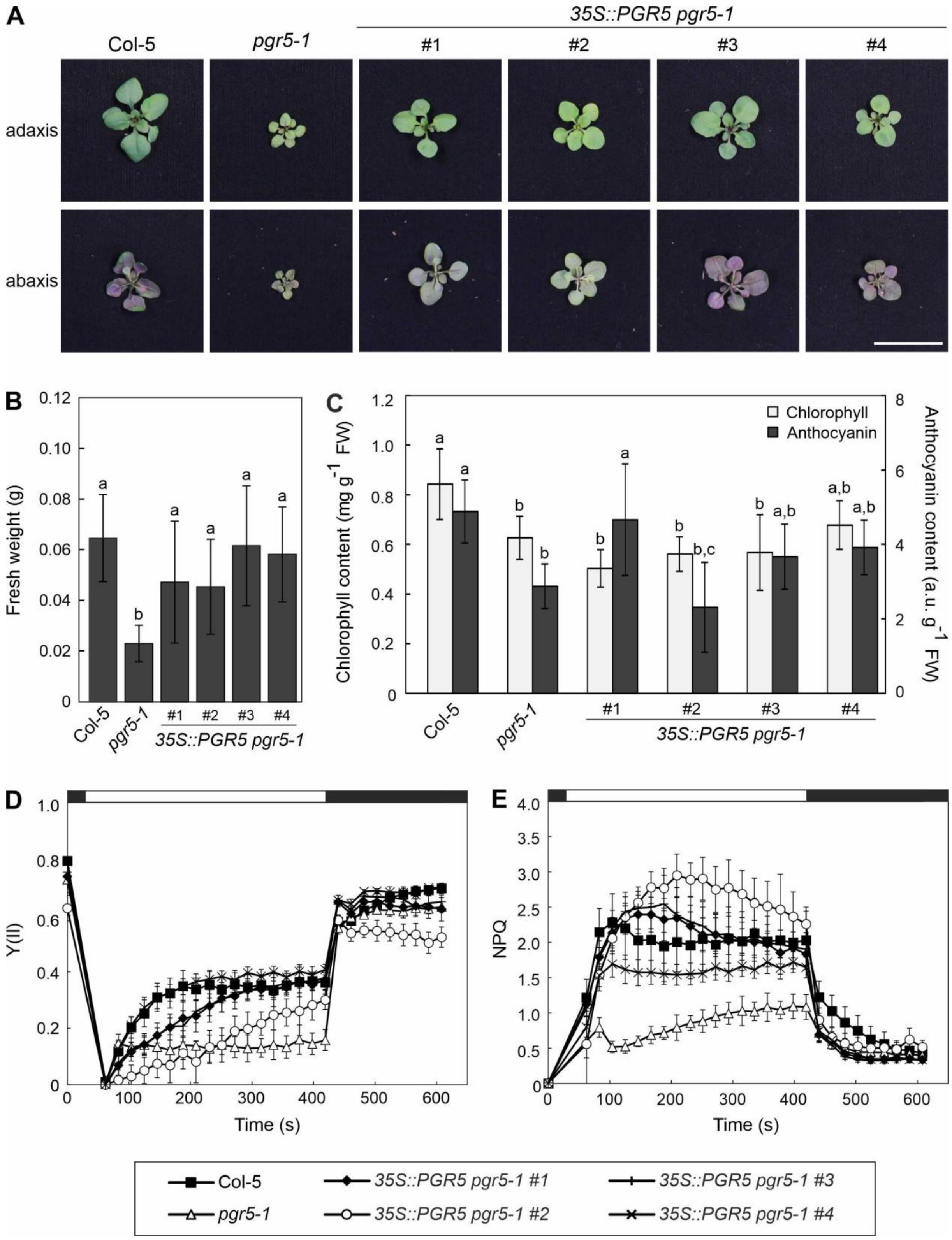

Supplemental Figure S7. Complementation of the *pgr5-1* mutant under high light conditions. **A**, Growth phenotypes of plants [wild-type (Col-5), *pgr5-1* and four different *pgr5-1* lines overexpressing PGR5 (*35S::PGR5 pgr5-1*, #1-4)] grown under high light (HL) conditions (16 h light / 8 h dark, 500 µmol photons m^{-2} s^{-1}) for 2 weeks. **B**, Fresh weight of plants grown as in (**A**). **C**, Chlorophyll and

anthocyanin contents of plants grown as in (A). Averages of at least three replicates are shown. Error bars represent standard deviations and different letters on error bars represent statistical difference between the lines ($p < 0.05$) by Tukey's test. **D,** Photosystem II quantum yield, Y(II), based on chlorophyll fluorescence monitored in dark-adapted plants grown as in (**A**). Plants were illuminated for 6 min with actinic light (400 μmol photons m^{-2} s^{-1}, white bar) and followed by a dark period of 3 min (black bar). Saturating pulses were applied every 20 s. **E**, NPQ corresponding to the measurements shown in (D). Averages of at least 7 plants from two independent experiments are shown. The scale bar at the bottom corresponds to 2 cm for all images in this panel.

Supplemental Table S4. Chlorophyll contents of wild-type (Col-0 and Col-5) and mutant (*pgrl1ab*, *pgr5-Cas#1*, *pgr5-Cas#2* and *pgr5-1*) plants grown under long-day (LD) and short-day (SD) conditions for 3 and 6 weeks, respectively. Averages of at least six replicates are shown ± standard deviations. There were no statistical difference between the lines by Tukey's test ($p < 0.05$).

	**Col-0**	***pgrl1ab***	***pgr5-Cas#1***	***pgr5-Cas#2***	***pgr5-1***	**Col-5**
**LD**	0.140 ± 0.009	0.157 ± 0.037	0.151 ± 0.008	0.142 ± 0.009	0.113 ± 0.008	0.132 ± 0.010
**SD**	0.178 ± 0.001	0.167 ± 0.005	0.164 ± 0.031	0.180 ± 0.005	0.139 ± 0.11	0.167 ± 0.006

Supplemental Table S8. Oligonucleotide sequences used for gRNA, sequencing and cloning.

**Locus**	**Gene**	**Primer (5' to 3')**	**Notes**
At2g05620	*PGR5*	Fw: GGTGTAAGTCCAAGCAAGA<br>Rv: CGGATTAAGAGCTGATGTTG	Sanger-sequencing for the *pgr5-1* mutant (S130 point mutation)
At2g05620	*PGR5*	Fw: TGCCTACTAGGTTGCGTGTG<br>Rv: CTGCTTCGATTTCTGCAATAG	Sanger-sequencing for the *pgr5-Cas* mutants
At2g05620	*PGR5*	Fw: ATTGAGACTCTGGCTTGTTGCGG<br>Rv: AAACCCGCAACAAGCCAGAGTCT	gRNA to generate *pgr5-Cas* mutants
At2g05620	*PGR5*	Hind3-Fw: TGATTACGCCAAGCTTTGTCCTCCTTGAATATTGG<br>Sac1-Rv: GGGGAAATTCGAGCTCCTAAGCAAGGAAACCAAGC	*PGR5* cloning between HindIII and SacI sites of pBI121 binary vector

Supplemental Methods

Electrochromic Shifts (ECS) Measurements

ECS measurements were performed as described before (Schreiber and Klughammer, 2008) on single attached leaves and evaluated as the change in absorbance at 515 nm, recorded with the P515/535 emitter-detector module of the Dual-PAM-100 system (Walz, Effeltrich, Germany). The change in the proton motive force (*pmf*) across the thylakoid membrane (ECS_T) was estimated from the total amplitude of the P515 signal decay after a 15-min exposure to 340 µmol photons m^{-1} s^{-1} and subsequent shift to darkness. ECS_T was normalized to a single P515 turnover flash (ECS_{ST}), recorded after 30 min of dark adaptation prior to each measurement (Ruhle et al., 2021).

Seedling growth and photosynthetic measurements

Surface-sterilized Arabidopsis seeds were plated on one-half-strength Murashige and Skoog (½ MS) medium containing 1% (w/v) agar. After stratification for 2 days in darkness at 4 °C, plants were grown at 22 °C under long-day conditions (LD, 16 h light/8 h dark) using white light (100 µmol photons m^{-2} s^{-1}).

Chlorophyll a fluorescence of plants grown on ½ MS plates for 1 week and under LD conditions was monitored following the same IRC protocol described in "Materials and Methods", but applying 110 µmol photons m^{-2} s^{-1} of blue actinic light and using an Imaging-PAM spectrophotometer (Walz, Effeltrich, Germany).

Supplemental Literature

Schreiber U, Klughammer C (2008) New accessory for the DUAL-PAM-100: The P515/535 module and examples of its application. PAM Application Notes **1:** 10
